# Gas-Mediated Dynamic Structure Evolution of Bimetallic Alloy Catalysts

**DOI:** 10.3390/nano15231828

**Published:** 2025-12-03

**Authors:** Yafeng Zhang, Pengfei Du, Bing Yang

**Affiliations:** 1 New Energy Materials and Physics Laboratory, School of Physics, Ningxia University, Yinchuan 750021, China; zhangyafeng@nxu.edu.cn; 2 CAS Key Laboratory of Science and Technology on Applied Catalysis, Dalian Institute of Chemical Physics, Chinese Academy of Sciences, Dalian 116023, China; pfdu@dicp.ac.cn

**Keywords:** bimetallic alloy, reaction environment, dynamic structure, in situ electron microscopy imaging, structure-activity relationship, heterogeneous catalysis

## Abstract

Bimetallic alloys are widely used as heterogeneous catalysts due to their unique physico-chemical properties for improving catalytic reactions. Typically, the structures of alloy catalysts are inherently dynamic under gas environments, which plays a crucial role in their catalytic activity, stability and selectivity. One method of enhancing the catalytic performance of bimetallic nanomaterials is, therefore, to tune or control the surface structure of the nanomaterials, and tremendous progress has been made in this area in the past decade. In this review, we primarily focus on the dynamic structure evolution of binary noble metal alloy catalysts influencing their catalytic performance during the thermal catalytic reaction. First, we summarize the advantage of binary noble metal alloy catalysts and their structure correlation with catalysis. Then, we examine how the structure of precious-metal-based alloy catalysts evolves in response to varying gas environments and the resulting structures impacts on heterogeneous catalytic activity. Further, the advanced characterizing techniques, i.e., in situ scanning/transmission electron microscopy (in situ S/TEM) and near-ambient pressure scanning tunneling microscopy (NAP-STM) are outlined for visualizing these structural evolutions. Finally, we summarize the remaining challenges and outlooks for the future in this research field and offer the potential direction of rational design catalysts with high energy-efficient and sustainable catalytic processes.

## 1. Introduction

Metal catalysts play a crucial role in heterogeneous reactions [1,2,3,4,5]. In particular, the past decade has witnessed the explosion of research on noble metal (Au, Pt, Pd, Rh, Ir) catalysts in the fields, such as environmental pollution control [6,7,8,9], electrocatalysis [10], and energy and chemical industry [11,12,13,14,15]. However, noble metals are extremely scarce in the earth’s crust. Therefore, reducing the use of expensive metals while improving catalyst performance is a key research project. One effective means is to partly replace them with cheap transition metals to fabricate bimetallic catalysts. This allows for reduced use of precious metals, offering huge significant potential in various catalytic applications.

It was determined that, in bimetallic alloy catalysts, the distinct physico-chemical properties of each metal can enhance catalytic performance beyond that of corresponding monometallic catalysts [5,16,17]. As bimetallic catalysts possess a shared energy band structure of two-metal components, their surface electronic and geometric structures can be continuously tuned by altering alloy composition. Specifically, by tuning the distance between the metal’s d-band center and the Fermi level, their adsorption properties towards reactants and products can be modified, and thus affecting the catalyst’s activity, selectivity, and stability [18,19,20,21]. For example, during a methanol oxidation reaction (MOR), CO is generated and strongly adsorbed on the Pt catalyst surface to reduce the active sites, and thus affecting the catalytic activity and stability for MOR [22]. In the studies of PdPt and PtRu alloy catalysts, PdPt and PtRu alloy catalysts show a bifunctional active site for MOR [23,24]. The introduction of Pd(Ru) into Pt nanoparticles enables Pd(Ru) to form Pd-OH(Ru-OH) species through dissociating H_2_O, while Pt catalyzes MOR to produce Pt-CO. Then, Pd-OH(Ru-OH) species react with adjacent Pt-CO intermediates, producing CO_2_. These bifunctional sites in PdPt and PtRu alloy catalysts can result in the continuous exposure of Pt active sites, enabling a continuous MOR catalysis. In another case, Yoo et al. used Pt_3_M alloy catalysts to oxygen reduction reaction (ORR) [25], confirming the catalytic activity has the following relationship: Pt < Pt_3_Zr < Pt_3_Co < Pt_3_Ni < Pt_3_Y. This is attributed to the change in Pt d-band center by the addition of transition metals, affecting the adsorption and desorption of Pt sites for oxygen-containing intermediates.

It was previously demonstrated that the catalyst’s activity, selectivity, and stability vary depending on the structural types of alloy catalysts [16,26,27,28]. According to the structure of bimetallic nanocrystals, they can be classified into rare alloys including single atom alloys, solid solution alloys, intermetallic compound, and core–shell structures. In early studies, there were various mechanisms applied to reveal the structure–activity relationships, such as size effects, geometric effects, synergistic effects, ligand effects, stain effects, and combinations thereof [24,29,30,31,32,33,34,35,36,37,38,39].

Over the past decade, there has been growing recognition that catalyst structures are dynamic during reactions [40,41,42]. In particular, the gas-mediated dynamics of alloy structures have garnered significant attention [43,44,45,46]. During catalysis, reaction environments (temperature, gases, reactant and product molecules) typically result in the complex dynamic changes in catalyst structures. These changes include atomic migration and reconstruction on the catalyst surface, the generation, transformation, or disappearance of dynamic active sites, as well as oscillations in chemical states, sizes, and shapes. For characterizations of dynamic structures of catalysts, in situ techniques, particularly transmission electron microscopy (TEM) and scanning tunneling microscopy (STM) [47,48,49,50,51], have enabled a direct observation of microstructural changes in materials under reaction conditions; these structural variations further alter catalyst’s performances [41,52,53,54,55,56,57,58,59]. Therefore, investigating the structural evolution and performance responses of catalysts under reaction conditions are essential for identifying active sites, elucidating reaction pathways, and understanding the catalytic mechanism.

Since the structure of alloy catalysts is highly sensitive to the atmospheric environments of heterogeneous catalysis, thus this review will first provide a concise overview of how atmosphere environments (oxidizing atmosphere, reducing atmosphere and redox atmosphere) influence the structure of binary noble metal alloy catalysts. It will then focus on the progress of in situ imaging technologies and their thermocatalytic applications. Finally, it aims to offer cutting-edge perspectives on the in situ identification of dynamically evolving surface structures in bimetallic alloy catalysts during heterogeneous catalytic reactions.

## 2. Gas-Mediated Dynamic Structure Evolution of Alloy Catalysts

**Figure 1:**
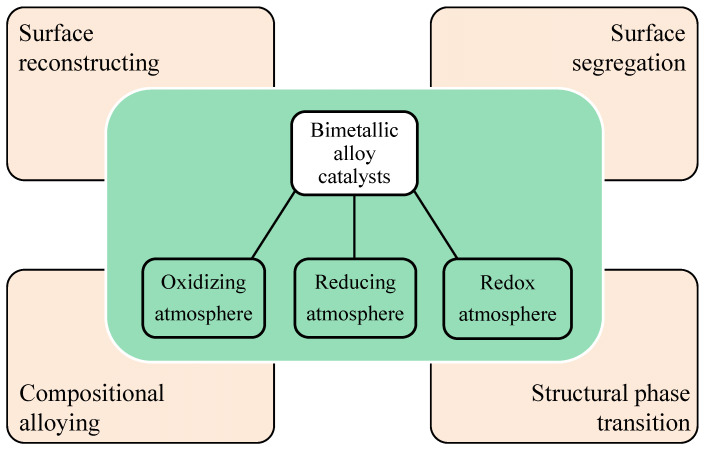
Schematic illustration of gas-mediated structure evolutions of bimetallic alloy catalysts.

Recent advances in atmosphere-mediated structure evolutions of binary noble metal alloy catalysts have been made (Figure 1, and Table S1), such as surface reconstructing, surface segregation, structural phase transition, and compositional alloying. Atmosphere-induced structure evolution is a dynamic process governed by the interplay of thermodynamics and kinetics. This phenomenon is driven by several underlying mechanisms [41,60,61,62,63]. For example, adsorbate-induced structural evolution of alloy catalysts. The chemisorption of gas molecules on the catalyst surface alters the surface electronic structure and surface energy [41,60,63]. To maximize (or minimize) this adsorptive interaction, the system drives components that form stronger (or weaker) bonds with the adsorbate to the surface. Another crucial mechanism is oxidation-/reduction-induced structural evolution. During this process, intensive changes in chemical potential under oxidizing or reducing atmospheres are the primary cause of structural transformation [61,62]. From the early monometallic catalyst studies to the current bimetallic catalyst studies, the atmosphere-mediated dynamic structure evolution of alloy catalysts has completely overturned the traditional understanding of material surface stability and has propelled the development of catalytic science.

### 2.1. Gas-Mediated Surface Reconstruction

Surface reconstructing of metal catalysts refers to the spontaneous rearrangement of atomic arrangement structure and chemical composition due to difference in the adsorption binding of gas molecule to different metallic component in alloys, alloying surface is typically subjected to surface rearrangement inspired by oxidating, reducing or oxidating- reducing atmosphere at a given temperature.

#### 2.1.1. Oxidation-Driven Surface Reconstruction of Alloy Catalysts

**Figure 2:**
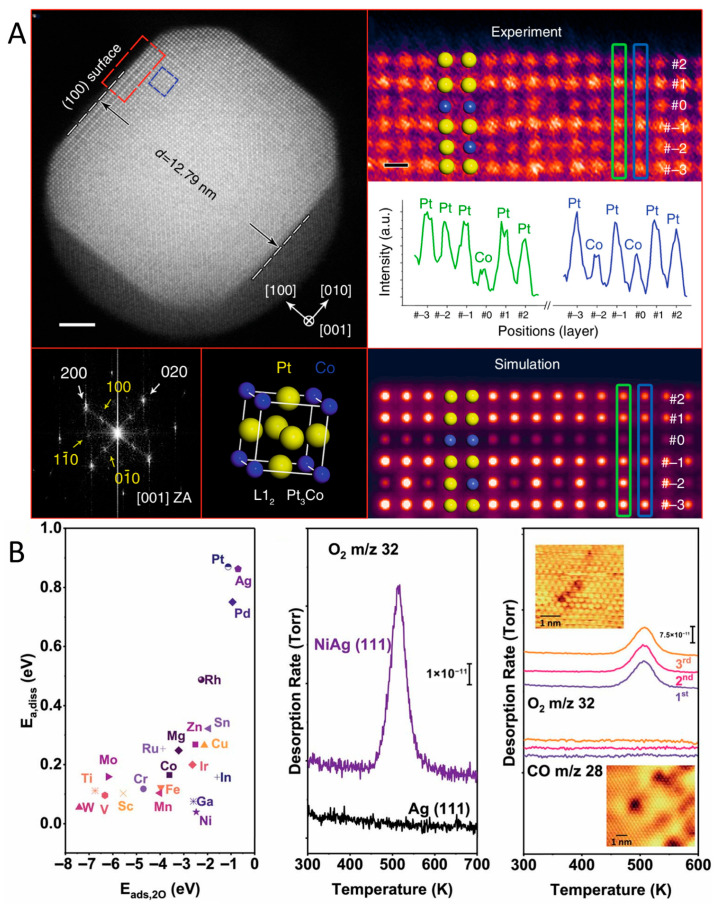
(**A**) In situ STEM results from a Pt_3_Co NP after oxygen annealing at 993 K [64]. (**B**) DFT calculations of the O_2_ dissociation barrier E_a,diss_ as a function of the adsorption energy of the resulting dissociated O atoms (E_ads,2O_) for various single-atom dopants in Ag(111) and O_2_ TPD traces, as well as sequential TPD traces (insets show STM images of samples) [45].

Oxidation-mediated atomic and valent rearrangements are the key driving forces for the dynamic reconstruction of alloy catalysts, which widely appeared in various alloy systems. For instance, in the case of Pt_3_Co alloying nanoparticles annealing at a temperature of 993 K for 30 min under the constant pressure of oxygen conditions, surface restructuring of PtCo alloys was unveiled using in situ STEM [64]. Experimental findings showed that oxygen annealing not only induces the initial disordered Pt_3_Co alloy to transform into ordered L1_2_ intermetallic Pt_3_Co alloys, but also promotes Pt surface segregation, forming a Pt-rich shell (Figure 2A). Density functional theory (DFT) calculations showed that this process follows an energetically favorable path. The ordered intermetallic structure of Pt_3_Co nanoparticles effectively prevents surface oxidation of Co. The Pt shell on the ordered intermetallic core can thus hinder Co segregation and protect the NPs from surface oxidation. In addition, several previous reports on Ag-based alloys also noted the oxygen-mediated surface restructuring phenomenon [45,65,66]. In a study of Ni-doped Ag alloys by Sykes et al. [45], the combination of the surface science technique and surface molecule adsorption revealed the influence of the oxygen atmosphere on Ni-doped Ag(111) surface, showing that the Ni-doped Ag surface can adsorb O_2_ and promote its dissociation (Figure 2B). Through temperature-programmed desorption (TPD) experiments, it was reported that when the Ni-doped Ag surface was exposed to O_2_ at 350 K, O_2_ dissociated and the oxygen atoms released could migrate to the Ag surface, forming adsorbed oxygen species. These oxygen species desorbed at around 520 K, which is approximately 70 K lower than the desorption temperature on the surface of pure Ag, indicating that Ni doping promotes the adsorption and release of O_2_. During this process, the scanning tunneling microscopy (STM) image found that under oxidizing conditions, Ni atoms were located on the Ag(111) surface, but under vacuum conditions, Ni atoms would migrate to the subsurface. This migration behavior indicates the dynamic changes in Ni under oxidizing and reducing conditions, further confirming the high dispersion and migration of Ni on the Ag surface. Using atom-resolved STM, Sykes’s group also indicated the dissociation behavior of O_2_ on the surface of AgCu alloy and the changes in surface structure at the atomic scale [65]. In the O_2_ atmosphere, the strong interaction between O_2_ and Cu atoms reduces the surface energy of Cu in the Ag layer. Therefore, the Cu atoms originally covered by the Ag layer are exposed. With the increase in O_2_ exposure, the increase in oxygen coverage will further promote the exposure of Cu atoms, forming the Cu_2_O structure. On the basis of a combination of ambient pressure X-ray photoelectron spectroscopy (XPS) and STM, an Ag-capped PdAg surface alloy was studied under the O_2_ atmosphere, confirming that exposure to 1 Torr of O_2_ at 400 K causes underlying Pd to resurface, and the resulting structure is stable at low pressures and low temperatures (300 K) [66]. Furthermore, in the case of PdAu alloys with dilute Pd, extended X-ray absorption fine structure (EXAFS) spectra proved that at high temperatures (673 K), O_2_ induces Pd atoms to segregate from subsurface layers to the surface, forming thermodynamically stable Pd-O structures through combination with oxygen [67].

#### 2.1.2. Reduction-Driven Surface Reconstruction of Alloy Catalysts

**Figure 3:**
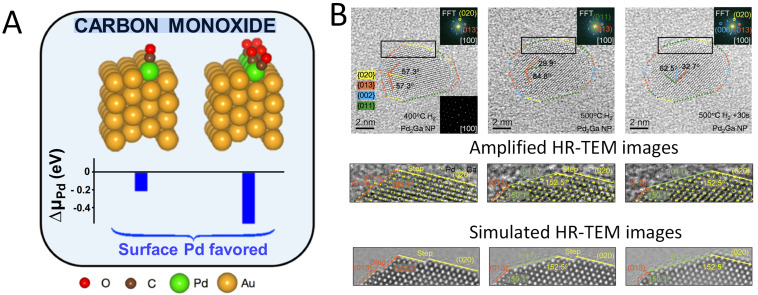
(**A**) Arrhenius plot of Pd dissolution observed in the temperature range of 313–453 K under a CO atmosphere [67]. (**B**) Atomic-scale structural identification of Pd_2_Ga alloy catalysts [68].

Reducing atmospheres, such as H_2_ or CO, have been shown be an efficacious tactic for modifying surface structure of alloy catalysts. For instance, it has been reported that H_2_ treatment significantly changes the structure of Pd_3_Cu alloy catalysts [58]. Using high-resolution STEM imaging, two types of surface segregation modes of Pd_3_Cu alloys were revealed for the first time. At 673 K, hydrogen reduction causes Cu enrichment on the catalyst surface. When the temperature rises to 873 K, Pd atoms move to the surface due to the stronger H_2_ adsorption on Pd, creating a Pd-rich layer. These structural changes alter the catalyst’s surface composition and electronic properties. Specifically, Cu-rich surfaces make the catalyst’s surface electron-rich, while Pd-rich surfaces make it electron-deficient. These electronic changes greatly affect the catalyst’s activity and selectivity in follow-up reactions. When PdAu alloys with dilute Pd was exposed to a H_2_ atmosphere, H_2_ promotes the dissolution of Pd into the subsurface of the Au matrix at high temperature (673 K), due to a reliable reason that Pd is more thermodynamically stable in the subsurface, when there is a single adsorbed H present. After CO treatment, more Pd atoms migrate to the surface in PdAu alloys (Figure 3A) [67]. A different study reported the surface restructuring of Pd_2_Ga alloys resulting from H_2_ molecules [68]. In situ TEM experiments were used to confirm the structuring crystal faces and the Pd placeholder on surface (Figure 3B). The authors found the phase transformation process of Pd_2_Ga nanoparticles from Pd to Pd_2_Ga in a hydrogen atmosphere, and the dynamic evolution of surface reconstruction of Pd_2_Ga alloys when the temperature rises from 673 K to 773 K (such as from (013)/(020) to (011)/(002) crystal planes). At a low temperature of 673 K, the surface of Pd_2_Ga is mainly composed of (013)/(020) crystal planes, and Pd atoms on the surface are arranged in a continuous trimer (Pd_3_) arrangement. At a high temperature of 773 K, the crystal plane is reconstructed into (011)/(002), and Pd atoms are isolated by the Ga atoms, forming isolated Pd_1_ sites.

Another approach to induce surface reconstruction of alloys are techniques using CO treatment. For instance, the surface dynamic reconstruction process of Pd atoms in the PdAu bimetallic catalyst under CO adsorption induction was uncovered through time-resolved temperature-programmed infrared reflectance absorption spectroscopy [69]. Below 320 K, CO adsorption can stabilize Pd single atoms on the surface. Above 320 K, CO desorption and Pd dissolution become the dominant processes. Using CO diffuse reflectance infrared fourier transform spectroscopy (CO-DRIFTS), Sykes et al. pinpointed the active geometric sites (single atoms or clusters) of PdAu bimetallic catalysts in a CO atmosphere [70]. At 303 K, after CO introduction, the CO adsorption peak (2076 cm^−1^) corresponding to isolated Pd atoms increased over time, while the peak at 2109 cm^−1^ decreased, indicating that CO drove Pd atoms from the Au bulk to the surface. Higher temperatures (343 K) lead to Pd aggregation. In addition, other studies on PdAu alloys also verified that the strong binding CO to Pd compared to Au results in more Pd atoms migrating to the surface [67,71].

#### 2.1.3. Redox Driven Surface Reconstruction of Alloy Catalysts

It has been reported that surface reconstructing could be modulated by using a cyclic oxidative/reductive process [72,73,74]. In situ DRIFTS spectra have shown that during cycles between CO_2_ and H_2_ flows, the redox process of surface Fe atoms was observed [72]. In situ DRIFTS showed that Fe^0^ on the surface of Pt-Fe catalyst was partially oxidized to Fe^n+^ in CO_2_. Fe^0^ generates CO by reducing CO_2_, and H_2_ reduces Fe^n+^ back to Fe^0^. In another case of PdAu alloys, in situ XAS and TEM have shown that the alternating atmosphere of O_2_/H_2_ can produce a change in structure of surface Pd sites [73]. Specifically, O_2_ treatment makes Pd more inclined to distribute on the catalyst surface. The local structure of Pd changed, the Pd-Pd coordination number increased slightly, and the Pd-Au coordination number decreased accordingly. The results show that O_2_ treatment promotes Pd surface enrichment to form trimers. H_2_ treatment causes Pd to dissolve into the Au matrix. After H_2_ treatment, the Pd-Pd coordination number decreases, while the Pd-Au coordination number increases. This indicates that H_2_ treatment makes Pd atoms more dispersed in the Au matrix, reducing the aggregation of Pd on the surface to form monomers or dimers. For a CuNi alloy studied using near pressure XPS [74], detailing surface segregation and structural reconstruction (O_2_ and H_2_) were disclosed under diverse temperatures and gas environments. The oxidative pretreatment at 973 K in O_2_ enriches the surface with Ni^3+^ species (Ni_2_O_3_/NiOOH) and drives the formation of Cu^2+^-enriched cores (Cu(OH)_2_ and CuO). Subsequent exposure to a H_2_ atmosphere at 673 K further enhances the segregation of metallic Ni to the surface.

#### 2.1.4. Dynamic Structure–Activity Relationship During Reaction

**Figure 4:**
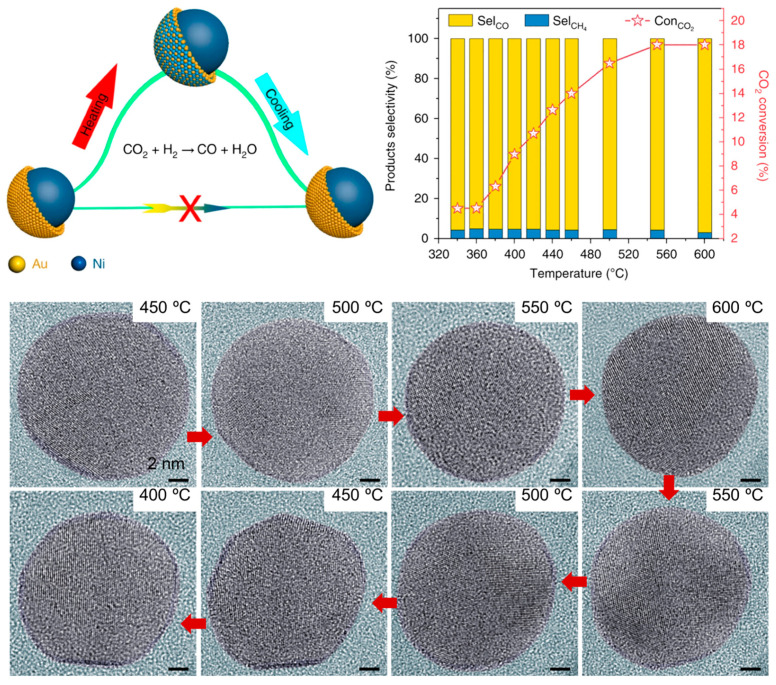
Structure–activity relationship of Ni-Au alloys during CO_2_ hydrogenation reaction [77]. All scale bar is 2 nm in ETEM images.

Under catalytic reaction conditions, the accurate identification of the real active structures of alloy catalysts is crucial for the dynamic understanding of structure–activity relationships and the precise design of highly active and stable alloy catalysts. It was previously demonstrated that surface structure of alloy catalysts is dynamic during reaction atmospheres [75,76]. In situ TEM and spectrum characterizations have shown the dynamic structural evolution mechanism of Ni@Au core–shell structured nanoparticles during CO_2_ hydrogenation reaction [77]. Traditionally, core–shell catalyst performance was thought to stem from its unique shell structure. However, this study shows that during reactions, the core–shell structure reversibly transforms into an alloy surface (Figure 4). Using environmental TEM (ETEM), Ni@Au nanoparticles were observed in situ in a CO_2_/H_2_ atmosphere at 9 mbar. The core–shell structure was stable at 723–773 K (450–500 °C) but became a NiAu alloy at 873 K (600 °C), with the core–shell structure reappearing upon cooling. Calculations based on DFT demonstrate that the whole process can be divided into two stages. In the first stage, the CO_2_ is hydrogenated at the Ni surface. The second stage is that the energetically favored adsorbed CO molecules diffuse from the Ni site to the Au sites. Meanwhile, CO drove Ni surface migration, achieving 95% of CO selectivity. But this high selectivity is not due to the initial core–shell structure. Instead, it is because the formed NiAu alloy surface, where Ni atoms enable easier CO_2_ activation for CO production. Similar surface reconstruction was also observed in Pd_3_Cu alloy catalysts during CO_2_ hydrogenation reaction [58]. It was found that in the reaction temperature range of 517–673 K, the Cu atoms in the Pd_3_Cu catalyst migrate from the metal particles to the SiO_2_ shell under the strong adsorption of oxygen-containing reaction intermediates and form small Cu clusters. This structural change leads to the formation of a unique core@shell@satellite (CuPd@SiO_2_@Cu) structure (denoted as Cu_3_Pd-673). The formation of this structure has an important influence on the activity of the catalyst. The specific manifestation is that the catalyst with enriched Cu surface shows low activity. This might be because the Cu enrichment on the surface weakens the dissociation ability of H_2_, and the enriched-electron Cu sites reduce the activation ability of CO_2_. By contrast, H_2_-produced Pd_3_Cu alloys with a Pd-rich surface at 873 K (denoted as Cu_3_Pd-873) show higher activity. This is because the presence of Pd atoms can effectively dissociate H_2_ and transfer the activated H atoms to the Cu surface through the electron overflow effect, thereby enhancing the hydrogenation reaction performance of the catalyst. In detail, within the temperature range of 517–673 K, the CO_2_ conversion of the Cu_3_Pd-873 catalyst was significantly higher than that of the Cu_3_Pd-673 catalyst. When the temperature rose from 517 K to 673 K, the CO_2_ conversion of the Cu_3_Pd-673 catalyst gradually increased from 0.3% to 8.6%, while the CO_2_ conversion rate of the Cu_3_Pd-873 catalyst significantly increased from 1.3% to 12.7%. This indicates that the Cu_3_Pd-873 catalyst exhibits higher activity in the CO_2_ hydrogenation reaction.

### 2.2. Gas-Mediated Compositional Segregation

Atmosphere-mediated phase segregation in alloys has become a cutting-edge research topic in materials science and surface chemistry. The segregation of elemental compositions occurs under non-equilibrium conditions caused by chemical potential differences in specific atmospheres, forming two-phase interfaces. This offers a new perspective for controlling catalyst dynamic behavior.

#### 2.2.1. Alloy Surface Segregation Under Heating Conditions

High-temperature treatment can cause the diffusions between heterogeneous atoms in alloys and thus result in variation in partial composition in alloys. Combined with in situ STEM and energy dispersive X-ray spectroscopy (EDX), Mirsaidov et al. observed the phase separation of AuRu alloy nanoparticles during heating increased from 623 K to 1023 K [78]. At the initial stage of phase separation, metastable phase decomposition of the alloy leads to the formation of small face-centered cubic (fcc) Ru clusters. When the temperature is increased, as the fcc Ru clusters grow larger, they undergo a phase transition to the more stable hexagonal close-packed (hcp) Ru structure. This transformation is attributed to the size-dependent competition between the interfacial and bulk energies of Ru domains. Under heating conditions of 973 K, the atomic diffusion of Au and Ru produced four Ru-rich domains. This finding showed that fcc AuRu alloy nanoparticles (containing about 85% Au and 15% Ru) will undergo phase separation, forming an fcc Ru intermediate phase from the fcc AuRu alloy, and finally forming a Ru phase with an hcp crystal structure.

#### 2.2.2. Oxidation-Driven Surface Segregation

**Figure 5:**
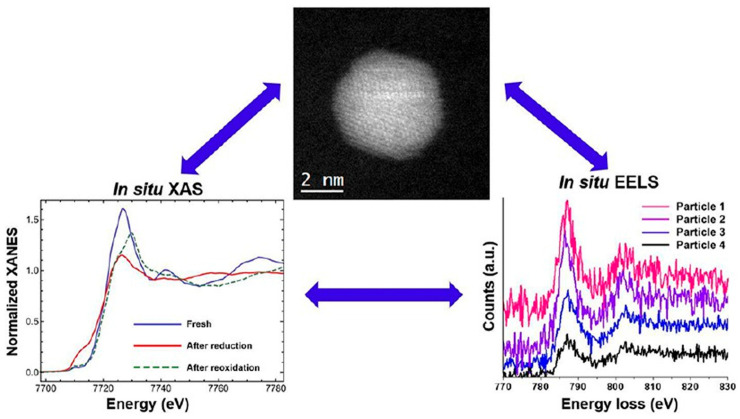
The dynamic structural characterizations of Co_2_Pt_3_ nanocatalysts under the O_2_ atmosphere [80].

Previously, the dependency of surface segregation of alloy catalysts on oxygen conditions that was used for the treatment of catalysts has been observed in AuCu and PtCo alloys [79,80]. Using ETEM, Park et al. revealed that in an O_2_ atmosphere, as the temperature increased, Cu atoms migrated from the interior of the AuCu nanoparticles to the surface and oxidized to form CuO_x_ species [79]. In this process, after O_2_ was introduced at room temperature, the amorphous coating on the surface of the AuCu nanoparticles was removed and Cu_2_O clusters were formed; as the temperature increased to 373 K, the Cu_2_O clusters transformed into CuO phases, and the Au-Cu nanoparticles became smaller and flatter. These results indicate that the oxygen atmosphere treatment induced atomic rearrangement and surface structural segregation of AuCu nanoparticles, forming a CuO_x_/Au heterostructure. The AuCu/SiO_2_ catalyst (O_2_-AuCu) pretreated with oxygen exhibited a significantly enhanced catalytic performance in CO oxidation reactions. Likewise, under the oxygen atmosphere, through in situ X-ray absorption spectroscopy (XAS) and STEM techniques (Figure 5), the dynamic structural evolution mechanism of Co_2_Pt_3_ nanocatalysts under the O_2_ atmosphere was systematically revealed [80]. STEM observations found that after O_2_ treatment, CoPt nanoparticles formed a distinct core–shell structure, with the shell being Co oxide (presumably Co_3_O_4_) and the core still being a CoPt alloy. Electron energy loss spectroscopy (EELS) analysis further confirmed the presence of oxygen in the shell, indicating that Co migrated to the surface during oxidation and formed an oxide layer, causing Pt atoms to be more confined inside the particles, thereby reducing the sites of Pt exposed on the catalyst surface.

#### 2.2.3. Reduction-Driven Surface Segregation

**Figure 6:**
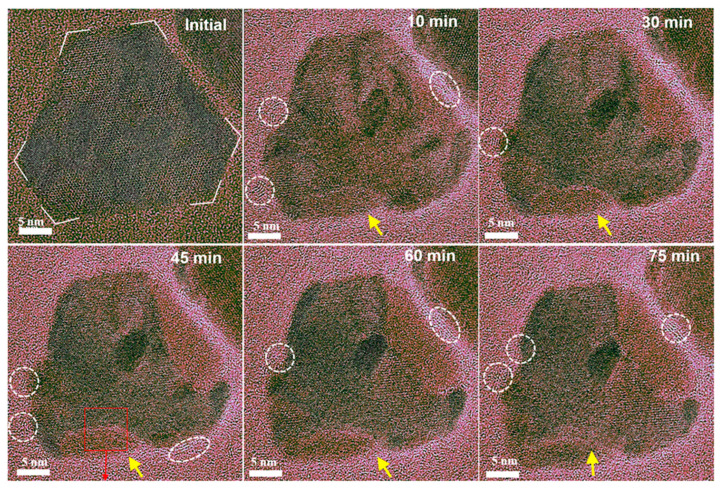
In situ TEM observation of the dynamic structure evolution of PtPb@Pt under 0.1 mbar CO gas [82].

How reduction treatments affect the compositional segregation for alloy catalysts has also been studied. For instance, using CO TPD and in situ CO polarization modulation infrared reflection absorption spectroscopy (PM-IRRAS) techniques to monitor the changes in surface composition and structure of Cu-Ni/SiO_2_ catalyst under changed CO pressure and temperature, Goodman et al. revealed that CO adsorption can induce Ni segregation on the surface of Cu-Ni/SiO_2_ bimetallic catalysts under normal pressure [81]. During this process, Cu is enriched on the surface of the CuNi alloys under ultra-high vacuum conditions. When the CO pressure was increased to the normal pressure, CO adsorption induced Ni segregation on the surface of the CuNi alloys. In situ PM-IRRAS results showed that under normal pressure CO conditions, the structure changed from Cu surface enrichment under UHV conditions to Ni surface enrichment under normal pressure CO, forming a surface Ni coating layer. Direct capture of the real-time structural evolution of PtPb@Pt nanosheets in CO environment has also been observed in situ TEM for the first time [82]. In a CO atmosphere (0.1 mbar, 623 K), the sharp corners of the hexagonal nanoplates gradually became rounded over time, and new lattice constants and orientations began to appear in some areas. After 10 min, these areas accounted for about 8%; after 75 min, the proportion increased to about 30% (Figure 6). High-resolution transmission electron microscopy (HRTEM) showed that the lattice spacing in these areas corresponds to the (220) crystal plane of Pt, indicating that CO induced the surface enrichment of Pt. During this process, lattice fringes generated by Pb-CO species were observed at the edge of the nanoplates, consistent with the structure of Pb(CO)_4_. This suggests that CO interacts strongly with Pb, pulling out single Pb atoms to form Pb(CO)_4_ species, which eventually accumulate to form an ultrathin Pb(CO)_4_ overlayer.

#### 2.2.4. Redox-Driven Surface Segregation

**Figure 7:**
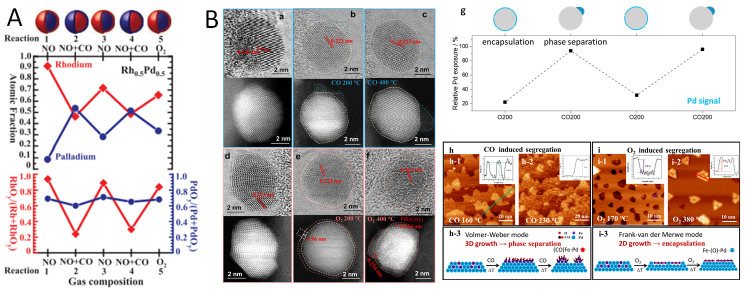
(**A**) The gas-dependent surface segregation of Rh-Pd nanoparticles [52]. (**B**) In situ TEM and STM characterizations of surface segregation of PdFe alloys under CO or O_2_ atmospheres [86].

As mentioned previously, surface segregation could be modulated by using a cyclic oxidative/reductive atmosphere [83,84]. In the study of AuPd alloy [83], the authors found that after treatment in the air atmosphere at 773 K, the size of AuPd nanoparticles increased from 2.5 to 3.5 nm to 5.5 nm. At the same time, Pd atoms migrated to the surface to form an Au-rich core and Pd shell structure. When the Pd content increases, the surface Pd is further oxidized to form a PdO shell. After H_2_ treatment at 773 K, PdO is reduced to metallic Pd, and a Pd-rich surface is formed on the surface. Using in situ TEM, Stach et al. revealed the dynamic structural evolutions of Au_0_._75_Pd_0_._25_ alloy nanoparticles under different gas environments (O_2_, H_2_, CO and CO_2_) and elevated temperature conditions. O_2_ environment (673 K, 1 bar) induced the Pd-enriched surface to form PdO, followed by H_2_ treatment leading to PdO being reduced to metallic Pd. However, replacing H_2_ with a CO atmosphere and increasing the temperature from room temperature to 473 K causes the destruction of the surface crystal structure of nanoparticles, the particle surface becomes irregular, and the surface roughness increases significantly. This is because the CO molecules adsorbed on the particle surface can change the surface energy, and thus cause surface segregation. When employing a CO_2_ atmosphere rather than CO, no obvious surface variation was observed for nanoparticles exposed to CO_2_ at 523 K, indicating that the interaction between CO_2_ and Pd or Au is weak, and it is difficult to induce changes in the surface structure of nanoparticles. Similar studies on the redox cycles of alloy catalysts have been performed under H_2_, CO, NO, and O_2_ conditions using a near-ambient pressure X-ray photoelectron spectroscopy (NAP-XPS) [52]. Using this surface technique by Somorjai et al., the elemental composition and chemical state changes on the surface of Rh-Pd nanoparticles are monitored under different NO, O_2_, CO and redox atmospheres (Figure 7A). Under oxidative conditions (100 mtorr NO or O_2_), Rh is almost completely oxidized in the surface layer and segregates to form a RhO-rich surface layer. Under a reducing atmosphere, Pd migrates to the surface, while Rh migrates to the interior. In the catalytic reaction (CO + NO), the chemical state of Rh also changes significantly, and RhO_x_ is reduced to metallic Rh^0^. The experiment also found that this structural change is reversible. When CO is removed and NO is retained, Rh migrates back to the surface and is oxidized again. Compared with Rh_0.5_Pd_0.5_, the shell of Pt_0.5_Pd_0.5_ nanoparticles is mainly composed of Pd under oxidative conditions. Under catalytic and reducing conditions, PdO_x_ is significantly reduced, but no obvious metal segregation is observed. In the studies on CuNi/SiO_2_ and CuCo/SiO_2_ catalysts [85]. In H_2_ or O_2_ atmospheres, Ni is concentrated on the surface of CuNi nanoparticles; in the O_2_ atmosphere, Cu is enriched on the surface. In the presence of O_2_, the oxidation state of Cu increases significantly, while the oxidation state of Ni does not change much. NAP-XPS results show that this surface enrichment phenomenon is reversible when re-exposed to H_2_, indicating that the surface structure of CuNi nanoparticles is sensitive to changes in atmosphere. In contrast, in an O_2_ atmosphere, Cu in CuCo alloys is irreversibly enriched on the surface, and the surface remains Cu-enriched even when re-exposed to H_2_. NAP-XPS results show that the proportion of Cu in the surface layer increases significantly after O_2_ treatment, and this enrichment does not reverse when re-exposed to H_2_, while in H_2_, part of the oxidation state is reduced, but the Cu enrichment still exists.

In addition, Zhang et al. employed in situ TEM and in situ scanning tunneling microscopy (STM) to unveil the micro-segregation behavior of PdFe alloys at the atomic scale (Figure 7B) [86]. The results indicated that the PdFe alloy phase formed under a hydrogen atmosphere exhibits a surface alloy structure of PdFe, suggesting that Fe atoms preferentially migrate to the sub-surface layers of Pd nanoparticles, thereby forming a Pd@PdFe core–shell configuration. During oxidative segregation, embedded two-dimensional FeO nano-islands were initially formed. With increasing temperature and pressure, these FeO nano-islands migrated from the embedded regions to the surface while maintaining a two-dimensional (2D) layered morphology due to strong metal–oxide interactions (SMOIs). In contrast, under a CO atmosphere, the strong interaction between CO and Fe led to the preferential formation of three-dimensional Fe nano-islands. As temperature and pressure rose, these islands underwent a structural transition into three-dimensional (3D) aggregated pinnacle-like features, revealing the microscopic pathway of phase separation in PdFe systems. Furthermore, the stronger binding of gaseous CO with Fe atoms stimulates Fe segregation out of the PdFe dilute alloys, resulting in 3D growth of Fe islands, whereas the dissociative adsorption of O_2_ results in 2D layer-by-layer growth of segregated FeO as encapsulation overlayers that bind strongly with the Pd surface underneath. By alternating between CO and O_2_ atmospheres, the segregation structure of Fe species demonstrate reversible characteristics.

#### 2.2.5. Dynamic Structure-Activity Relationship During Reaction

**Figure 8:**
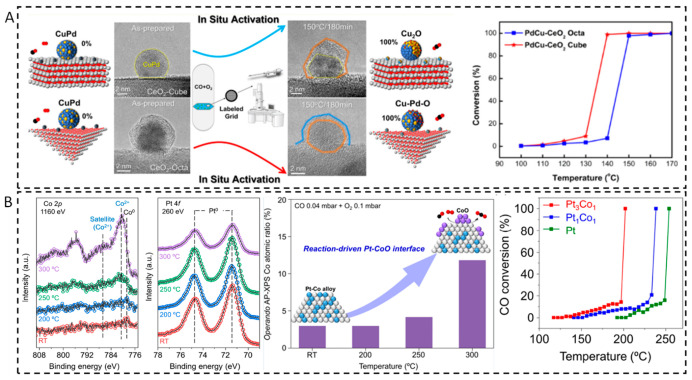
Structure–activity relationship of (**A**) PdCu/CeO_2_ catalysts [87] and (**B**) PtCo catalysts during CO oxidation [90].

Changes in the surface segregation of alloy nanoparticles during the real reaction have also been reported [87,88]. During a CO oxidation reaction, the structure of the PdCu nanoparticles is strongly related to the crystal plane of the carrier (Figure 8A) [87]. On the PdCu nanoparticles supported by CeO_2_ cubes (CeO_2_-C), the phase separation occurs, leading to a two-phase Cu_2_O/PdCu structure formation, while the nanoparticles supported on CeO_2_ octahedrons (CeO_2_-O), a uniform CuPdO_x_ phase is formed. For catalysts supported by CeO_2_-C, the Cu_2_O/PdCu interface formed can promote the efficient conversion of CO. While for PdCu catalysts supported by CeO_2_-O, the CuPdO_x_ phase formed shows a low activity compared to PdCu/CeO_2_-C catalyst. This was mainly attributed to the structural differences formed during reaction process. Using in situ TEM, similar study on PdCu alloy catalysts found that in an O_2_-rich atmosphere (CO oxidation reaction), PdCu alloys undergo phase separation to form Pd-CuO structures [88].

With in situ DRIFTS, H_2_-TPR, CO-TPR, EPR, and isotope labeling experiments, it has also been observed that PtCu alloys in PtCu/MgO catalysts were subjected to dynamic structural evolution in a CO oxidation reaction [89]. At low temperature (<423 K), CuO_x_ species are formed on the surface of PtCu alloys, which promotes CO oxidation. CuO_x_ species can provide abundant active oxygen species and significantly enhance the CO oxidation activity at low temperatures. At high temperatures (>423 K), PtCu alloys synergize with surface CuO_x_ species to promote CO oxidation. In follow-up studies, NAP-XPS was utilized to reveal surface segregation of PtCo alloys under the conditions of the CO oxidation reaction [90]. NAP-XPS results showed that as the temperature increases (Figure 8B), Co gradually oxidizes from a metallic state into CoO and finally forms CoO. Pt remains in a metallic state throughout the reaction, indicating the formation of a Pt-CoO interface. The as-formed Pt-CoO interfaces significantly enhanced their catalytic activity for CO oxidation compared to the single-metal Pt and Co catalysts. Similarly, dynamic structural changes in heterostructured Ni-Rh nanoparticles were also found in the CO oxidation reaction [91]. In situ TEM revealed that under oxygen-rich conditions (O_2_-rich), the Ni core began to become empty at 673 K, and the overall size increased, as well as part of the Ni was converted to NiO. While under oxygen-poor conditions, the contrast of Rh nanoparticles gradually decreased above 523 K, and the overall contrast was more uniform at 673 K, indicating that the Rh nanoparticles alloyed with the Ni core to form a NiRh alloy surface. The Ni-Rh nanoparticles under oxygen-rich conditions indicate high catalytic activity at low temperature, which is much higher than that of pure Ni and Rh nanoparticles. Under oxygen-poor conditions, the activity of NiRh nanoparticles decreased. This is because the surface of the NiRh alloy is more susceptible to CO adsorption, resulting in CO poisoning, which reduces the catalytic activity.

### 2.3. Gas-Mediated Dynamic Phase Transition

Atmosphere-mediated structural phase transition in alloys holds great importance in condensed matter physics, materials chemistry, and catalytic science. In this process, alloy materials, when exposed to specific atmospheres, undergo a transformation in their crystal structure. This is triggered by differences in chemical potential, the diffusion barriers, and interfacial energy of different elements, resulting in the atomic rearrangement that is entirely different from the original crystal structure.

#### 2.3.1. Alloy Phase Transition Under Vacuum or H_2_ Atmosphere

**Figure 9:**
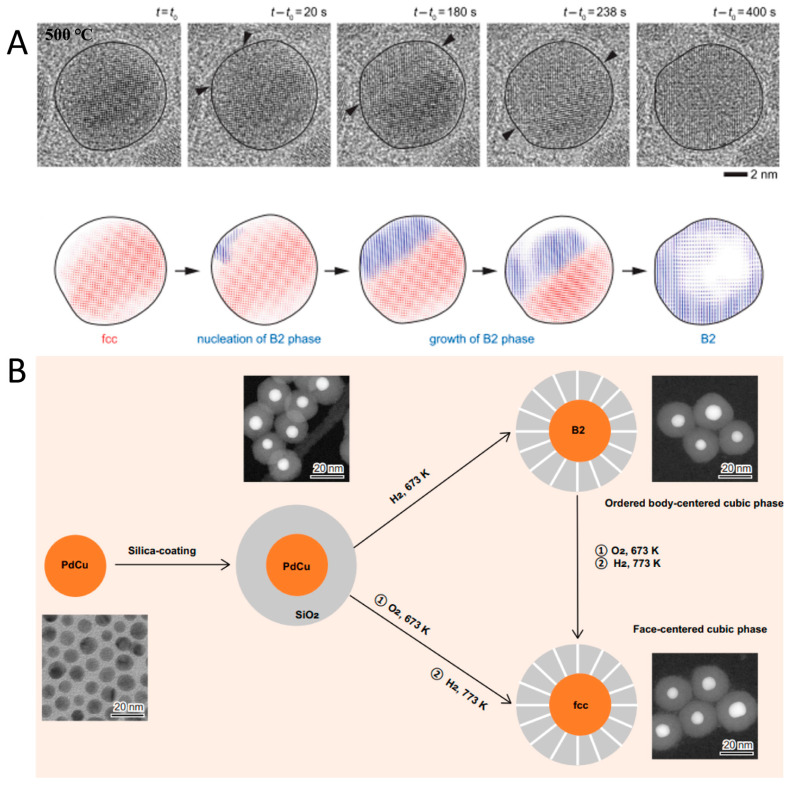
(**A**) In situ TEM characterization of a phase transition of fcc PdCu alloy into B_2_ alloy [92]. (**B**) A schematic diagram of tuning crystal-phase of the PdCu catalysts [93].

It was previously demonstrated that alloy phase transformation can emerge under vacuum or an inert atmosphere [92]. Employing in situ heating TEM under vacuum conditions (Figure 9A), Utkur Mirsaidov et al. elucidated the dynamic mechanism of the fcc-to-bcc phase transition in single-crystalline PdCu alloys [92]. They observed that the bcc phase originates at the edges of fcc nanoparticles and propagates inward to form an exclusive bcc phase. At 773 K, metastable fcc nanoparticles transform into the ordered B_2_ bcc phase. Similar fcc-to-bcc phase transition in PdCu alloys was also realized by the effective method reported by Shen et al. [93]. The authors subjected the initial PdCu colloid to hydrogen treatment at 673 K, inducing a phase transformation to the ordered B_2_ bcc structure (Figure 9B). This process enabled the formation of an ordered arrangement of Pd and Cu atoms within the nanoparticle. Additionally, to transform the B_2_-phase PdCu nanoparticles into the disordered fcc phase, the authors first treated them with oxygen at 673 K, followed by the reduction with hydrogen at 773 K. In the resulting fcc phase, Pd and Cu atoms are randomly mixed without forming ordered Pd-Cu bonds.

In a study using in situ XRD, it has been reported that the changes in the PdCu crystal structure during annealing under a N_2_ atmosphere reveal the phase transformation process from fcc to bcc. At low temperature (<573 K), the heat annealing treatment mainly forms PdCu with an fcc structure [94]. When temperature increases from 573 K to 773 K, the heat annealing treatment causes the PdCu to transform from the fcc phase to the bcc phase. When the annealing temperature reaches 773 K, the bcc phase becomes the dominant structure. Further increasing the annealing temperature (>773 K) will lead to a reduction in the bcc phase and the reappearance of the fcc phase, accompanied by particle aggregation and size increase. In addition, it has been reported that the structural conversion between disordered and ordered alloys in different gases conditions [95]. Subjecting disordered PdCu alloys (D-PdCu/C) to high-temperature treatment in a hydrogen atmosphere promotes the formation of atomically ordered structures. Annealing the D-PdCu/C catalyst in hydrogen at 773 K yielded the ordered PdCu/C catalyst. However, in a follow-up study [96], under a N_2_ atmosphere, the atomically ordered Pd_3_Bi was subjected to thermal annealing at 773 K to transform it into disordered Pd_3_Bi alloys.

#### 2.3.2. Dynamic Structure–Activity Relationship During Reaction

It was generally accepted that there is a close relationship between structural phase changes and catalytic performance for alloy catalysts. Adjusting the alloy’s phase structures can effectively optimize catalytic performance. This offers new methods for developing high performance catalysts. To investigate the relationship between alloy structural ordering and catalytic performance, the work by Zheng et al. [97], provides compelling experimental evidence. They first synthesized disordered PdCu alloy nanoparticles via a chemical reduction method. Subsequently, annealing these particles under a H_2_ atmosphere successfully induced a structural phase transition, resulting in the formation of an atomically ordered PdCu structure. This transition from disorder to order significantly enhanced the alloy’s catalytic activity. Within the ordered PdCu structure, Cu and Pd atoms show a specific arrangement, generating a high density of Cu-Pd pairs. These Cu-Pd pairs act as catalytic active sites, significantly boosting the surface coverage of the CO intermediate. In the electrocatalytic CO reduction reaction, experimental testing revealed that at an applied potential of −1.03 V (vs. the reversible hydrogen electrode, RHE), the ordered CuPd catalyst achieved a partial current density for acetate of 425 mA cm^−2^. This value is substantially higher than the performance observed for pure Cu nanoparticles and other samples. These results clearly demonstrate that the ordered structure of CuPd significantly boosts the activity for acetate production from CO_2_ reduction. This work strongly validates the critical role of structural ordering in enhancing the catalytic performance of alloy catalysts.

Through thermal annealing of fcc PdCu/C under an Ar/H_2_ atmosphere, Huang et al. achieved a phase transformation from an fcc structure to the bcc structure. Complete conversion to the bcc phase occurred at 623 K [98]. This change in crystal phase significantly affected the electrocatalytic performance for the nitrogen reduction reaction (NRR). The bcc PdCu catalyst exhibited superior electrocatalytic activity, achieving a NH_3_ production rate of 35.7 mg h^−1^ mg^−1^_cat_ with a Faradaic efficiency (FE) of 11.5% at −0.1 V versus the reversible hydrogen electrode (RHE). Importantly, it demonstrated high selectivity, with no hydrazine (N_2_H_4_) detected. In contrast, the fcc PdCu showed a substantially low NH_3_ yield, highlighting that the alteration in crystal phase structure markedly enhances the catalyst’s activity. Furthermore, the bcc PdCu catalyst exhibited excellent long-term stability. After five consecutive electrolysis cycles, no significant decay in current density was observed, and the NH_3_ production rate and Faradaic efficiency showed only marginal changes.

**Figure 10:**
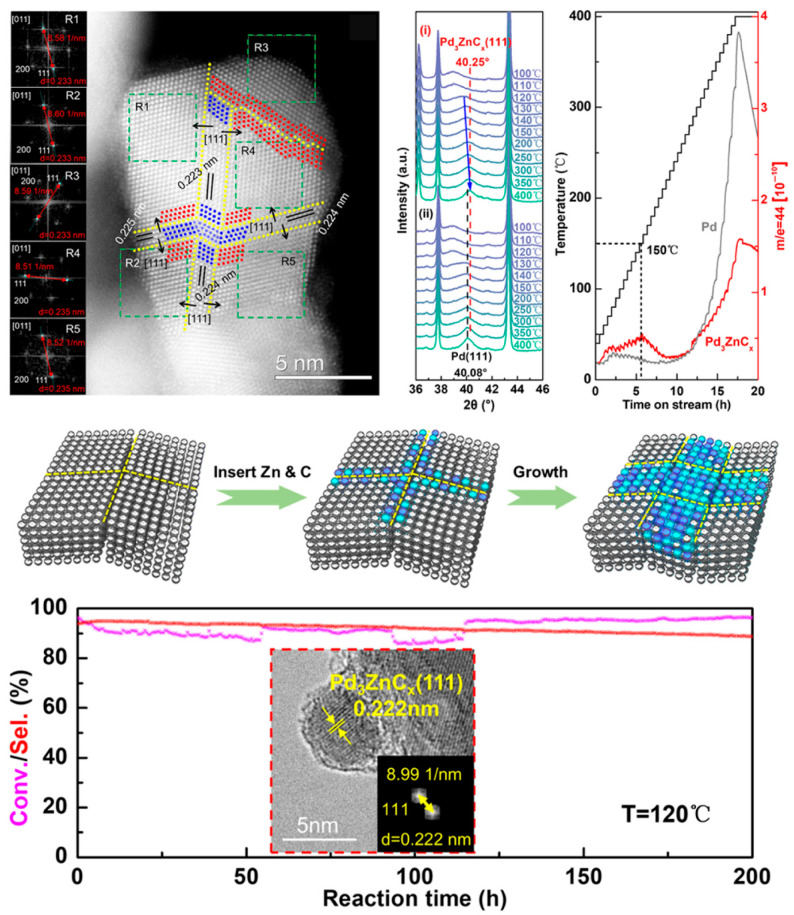
Syngas-mediated formation of PdZnC_x_ intermetallic carbide phase for boosting selective hydrogenation of acetylene [99].

In another case [99], Zhang et al. employed in situ XRD, XPS, and ETEM techniques to investigate in detail the temperature-dependent phase evolution of the PdO/ZnO/Al_2_O_3_ catalyst under syngas (CO/H_2_). At low temperatures (<393 K), PdO is initially reduced to β-PdH_x_ hydride. As the temperature increases to 393–623 K, β-PdH_x_ gradually decomposes into α-PdH_x_, and a surface-alloyed intermediate begins to form at the grain boundaries. Upon exceeding 623 K, this intermediate state facilitates the continuous incorporation of zinc and carbon, ultimately leading to the formation of a well-defined Pd_3_ZnC_x_ intermetallic carbide phase. This intermetallic carbide phase demonstrates exceptional catalytic performance in the selective hydrogenation of acetylene. It maintains high selectivity (>90%) towards the desired C_2_H_4_ product and exhibits superior long-term stability. These performance enhancements are primarily attributed to the optimized electronic structure and geometric advantages of the Pd_3_ZnC_x_ phase (Figure 10).

### 2.4. Gas-Mediated Compositional Alloying

Atmosphere-mediated alloying in bimetallic catalysts refers to bimetallic nanoparticles undergoing atomic-scale mixing under specific reducing environments, forming an alloy phase with homogeneously distributed compositions. This atmosphere-mediated alloying process can significantly alter the catalyst’s electronic structure and geometric configuration. Consequently, it provides a novel approach for designing and optimizing new generations of highly efficient alloy catalysts.

#### 2.4.1. Reduction-Driven Alloying of Bimetallic Nanoparticles

Changes in the composition distribution of Pd-based bimetallic nanoparticles upon hydrogen have been reported by Lu et al. [100]. In situ characterizations including XPS, EXAFS, XRD and STEM were used to study the changes in the surface structure of Pd based catalysts in a H_2_ atmosphere. At 573 K, the hydrogen reduction of InO_x_-coated PdCu catalysts prompts InO_x_ to move from the SiO_2_ support to the PdCu nanoparticle surface, thus creating PdIn alloys with Pd surface enrichment. Using similar characterizations, the structural changes in Pd@Ga_2_O_3_/Al_2_O_3_ catalyst were also revealed under a H_2_ atmosphere, showing that the Pd/Ga_2_O_3_ catalyst mainly forms the Pd_2_Ga phase, while the Pd@Ga_2_O_3_/Al_2_O_3_ catalyst forms both Ga-rich PdGa_5_ and PdGa alloy phases [101]. In addition, it was found that the structure of Pd@Au core–shell nanoparticles changes significantly under different CO pressures [102]. Under low CO pressure (below 10^−3^ mbar), CO adsorption absorbs predominantly on the Pd core. As CO pressure increases, CO adsorption triggers the diffusion of Pd atoms from the core towards the surface of the Au shell, leading to the formation of a Pd-Au alloy layer. When CO pressure exceeds 10^−3^ mbar, Pd atoms driven by CO adsorption migrate further to the shell surface, resulting in the formation of Pd dimer structures.

On the other hand, thermal annealing in a H_2_ atmosphere promotes nanoparticle alloying, such as Pt-based and Au-based catalysts [103,104,105,106]. Wang et al. proposed a general strategy for synthesizing high-entropy alloys (HEAs) under mild conditions by leveraging strong metal–support interactions (SMSIs) [104]. This work revealed that SMSIs promote the formation of multi-component alloys (MCAs) at significantly lower reduction temperatures (673–873 K), such as the synthesis of compositionally complex PtPdCoFe HEAs supported on anatase TiO_2_. Using S/TEM studies of Pt@Ru core–shell catalysts treated by H_2_ atmospheres [103], Shao et al. revealed that at 373 K, the Pt precursor is preferentially reduced to Pt nanoparticles, while most of Ru precursors remain as atomically dispersed species. At 573 K, a well-defined Pt@Ru core–shell structure forms, with Ru encapsulating the Pt core. As the annealing temperature is further increased to 723 K and 873 K, Pt atoms diffuse outward to the particle surface, ultimately forming a disordered PtRu alloy. Shen et al. revealed distinct structural transformations in Pt/TiO_2_ catalysts under H_2_ atmosphere at 773 K, which depend on Pt particle size [105]. For 2 nm Pt particles, a bulk Pt−Ti alloy formed. In contrast, 4–6 nm Pt particles formed a Pt_3_Ti intermetallic compound, forming a Pt_3_Ti@Pt core–shell structure. When the reduction temperature was further increased to 873 K, Pt particles in the 2–6 nm size range were fully converted into well-crystallized Pt_3_Ti intermetallic compound structures. In a follow-up study [106], Cu@Au core–shell nanowires undergo a disorder-to-order phase transformation upon thermal annealing in a H_2_/Ar atmosphere, ultimately forming Cu_3_Au intermetallic compound nanowires. The results demonstrate that within the temperature range from 413 K to 593 K, the nanowires progress through three distinct stages. Below 473 K, partial alloying of Cu and Au predominates; a mixture of tetragonal CuAu (L1_0_ phase) and Cu phase emerges between 473 K and 493 K; above 493 K, the Cu_3_Au phase (L1_2_ structure) becomes predominant.

#### 2.4.2. Dynamic Structure–Activity Relationship During Reaction

**Figure 11:**
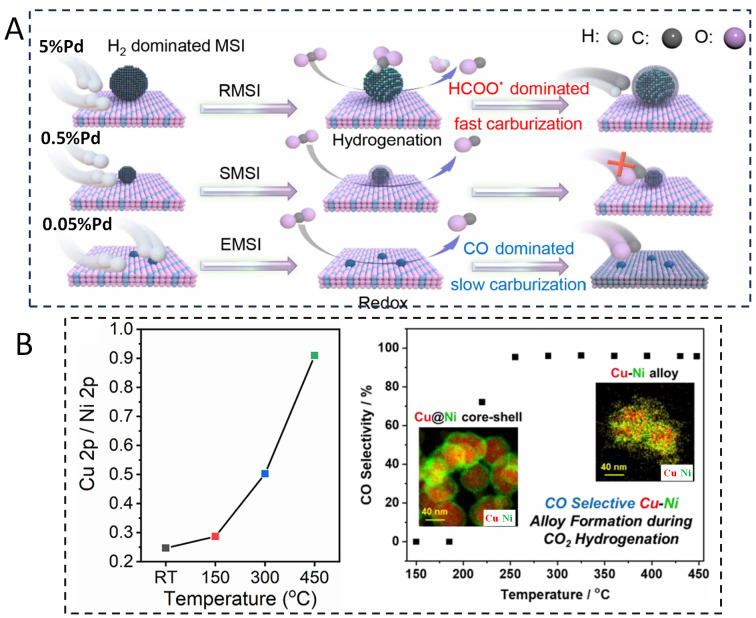
(**A**) The schematic diagram of size-dependent domino effect on Pd/FeO_x_ catalysts during CO_2_ hydrogenation reaction [107]. In this case, RMSIs are evidenced by the structural transformation of the 5%Pd/FeO_x_ catalyst from Pd to a Pd_3_Fe alloy under reaction conditions. SMSIs are indicated by the formation of a Pd@FeO_x_ core–shell structure. EMSIs are defined by the electron transfer between Pd single atoms and the FeO_x_ support. * represents absorbate. (**B**) Structure–activity relationship of Cu-Ni catalyst during CO_2_ hydrogenation reaction [108].

In a recent study on Pd/FeO_x_ catalysts [107], in the CO_2_ hydrogenation reaction, Du et al. revealed the dynamic evolution process of the Pd-FeO_x_ catalyst, particularly highlighting the “domino effect” between the reaction network and the structural reorganization of the catalyst (Figure 11A). The 5% Pd/FeO_x_ catalyst was studied with in situ XRD, XPS, DRIFT, and STEM during reaction. The results indicated that Pd particles with larger sizes (4–5 nm) induce a reactive metal–support interaction (RMSI), leading to the formation of a Pd_3_Fe alloy. This alloy formation significantly alters the catalyst’s surface composition, promoting a shift in the reaction pathway from the redox pathway involving direct CO_2_ dissociation to the hydrogenation pathway proceeding through a HCOO^−^ intermediate, increasing the yield of CO. Ryoo et al. reported the dynamic evolution behavior of Cu-Ni catalysts during CO_2_ hydrogenation reaction [108]. Utilizing characterization techniques such as in situ XPS and DRIFTS, researchers discovered that under reaction conditions, Cu atoms migrate from the core to the surface. This migration transforms the initial Cu@Ni core–shell structure into a Cu-Ni alloy structure. NAP-XPS experiments revealed that at room temperature, the surface layer of Cu_0.5_Ni_0.5_ nanoparticles is Ni-enriched, exhibiting a Cu/Ni atomic ratio of 0.25. As the reaction temperature progressively increases, the Cu/Ni atomic ratio rises to 0.91, indicating significant surface segregation of Cu from the core to the surface and the creation of new active sites (Figure 11B). Following this structural alloying, the catalyst’s surface adsorption capacity for reaction intermediates is altered. For instance, in CO_2_ hydrogenation, the Cu-Ni alloy surface more effectively adsorbs and activates CO_2_ molecules, facilitating their conversion into adsorbed CO_2_ species. Concurrently, the dissociation and adsorption of H_2_ are enhanced, enabling more hydrogen atoms to participate in the reaction, thereby boosting overall catalytic activity. A further example of Pd-Pt alloying has been reported [109]. In situ XRD results have shown that at a reduction temperature of 523 K (250 °C), the Pd-Pt/In_2_O_3_ catalyst exhibited significant structural changes, indicating the formation of a PdPt alloy. Under CO_2_ hydrogenation reaction, in situ XRD results showed that the PdPt alloy phase remained stable at 573 K (300 °C), indicating that this alloy structure has good stability during the reaction process.

## 3. In Situ Characterizations for Dynamics of Bimetallic Alloys

In the fields of materials science, energy, and catalysis, the resolution of numerous critical questions remains constrained by the limitations in detection thresholds, and instrumental sensitivity. These persistent challenges, however, can be fundamentally addressed through advanced methodologies in electron microscopy and surface science. Recent breakthroughs in in situ aberration-corrected transmission electron microscopy (in situ TEM) and surface probe techniques such as in situ or near-ambient pressure scanning tunneling microscopy (in situ/NAP-STM) deliver the experimental foundation for rationally designing nanoscale materials that tune performance, and for revealing the dynamic mechanisms that govern catalysis.

### 3.1. In Situ TEM Characterization

The wave-like nature of electrons, proposed by French physicist Louis de Broglie in 1923, provided the theoretical foundation for using electrons rather than visible light to probe the microstructure of matter [110]. Building on this insight, German scientists Max Knoll and Ernst Ruska constructed the first TEM in 1932, achieving a resolution better than 2 nm [111]. This breakthrough opened an era of rapid TEM development. So far, high-resolution TEM routinely delivers 0.2 nm resolution, and the advent of spherical-aberration correctors has pushed the limit below 80 pm, enabling direct, atom-scale visualization of material structures.

Recent advances in environmental/in situ aberration-corrected transmission electron microscopy (in situ TEM) and dedicated in situ specimen holders allow researchers to visualize, at atomic or molecular resolution, how a material’s microstructure evolves under realistic and multi-physical stimuli such as gases, heat, liquids, light, electric fields, and mechanical stress. Catalysis has emerged as one of particular application areas [42,46]. By engineering “micro-reactors” inside the microscope column, the entire catalytic process can be monitored in real time, thus mimicking operating conditions and yielding comprehensive and unambiguous insights into complex reaction pathways.

**Figure 12:**
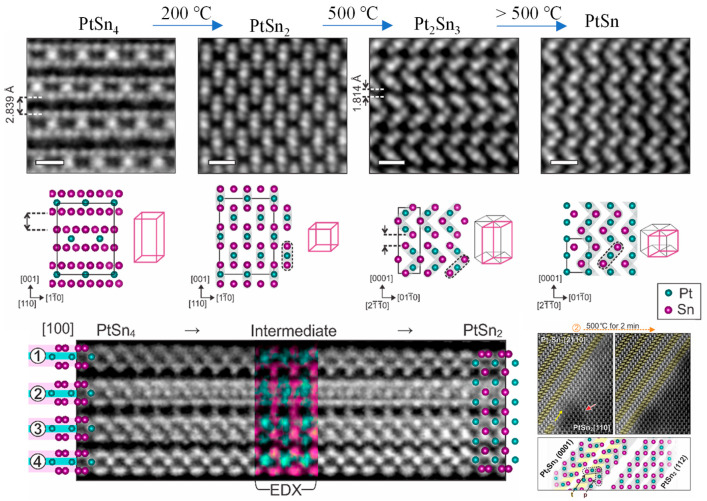
Atomic-resolution HAADF-STEM images of PtSn_4_ to PtSn multi-level phase transformation [117]. Scale bars are 0.5 nm.

Over the past decade, many research groups have exploited in situ TEM to track gas- mediated structural dynamics of supported metal catalysts. The dynamic structural evolution of nanocrystals has been captured in situ, such as sintering and agglomeration, redispersion, phase segregation, alloying, and reconstruction phenomena [75,100,112,113,114,115]. These observed unconventional behaviors advance the study of structure–performance relationships in catalysis. In a typical example [116], Wang et al. delivered the anatase TiO_2_ featuring a distinctive (1 × 4) reconstruction on its (001) facet. By leveraging highly ordered rows of 4-coordinate Ti atoms as active sites, they successfully visualized the dissociation of water molecules and the water–gas shift reaction process. Using in situ heating atomic-resolution STEM [117], Yun et al. have unveiled the atomic behavior and structural evolution of Pt−Sn alloys during distinct phase transformation processes (Figure 12). Their observations reveal that during the phase transition from PtSn_4_ to PtSn_2_, an intermediate phase (PtSn_2_) begins to form as the temperature increases to 473 K. When the temperature reaches 773 K, the transformation from PtSn_2_ to Pt_2_Sn_3_ is initiated, with Pt_2_Sn_3_ nucleating on the surface of PtSn_2_ and subsequently growing along specific crystallographic directions. Above 773 K, PtSn_2_ undergoes an isotropic phase transformation into PtSn species.

### 3.2. NAP-STM Characterization

In 1981, Binning, Rohrer et al. at the Zurich Institute developed the scanning tunneling microscopy (STM) based on quantum tunneling principles [118]. This breakthrough not only heralded humanity’s entry into the atomic-scale era of material characterization but also enabled atomic-scale material manipulation and single-atom control. The technique exhibits three defining advantages, such as atomic-scale resolution (~0.01 nm), direct visualization of local surface microstructures, and operational versatility across diverse environments (vacuum, ambient air, room temperature). This achievement provided the first real-time observation of individual atoms’ spatial arrangements on material surfaces and their associated electronic behaviors. It has generated profound implications for fundamental research in surface science, materials engineering, and so on.

**Figure 13:**
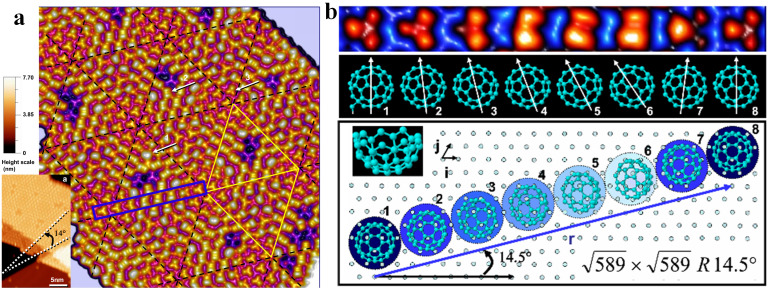
(**a**) C_60_ island on the surface of Au (111) [123], the superstructures on the island are composed of rhombic unit cells with 49 of molecules (20 nm × 20 nm, U = 1.5 V, I = 0.1 nA). (**b**) STM image of the C_60_ molecules and schematic diagram of their adsorption orientations located in the blue rectangle in (**a**).

Breakthroughs in NAP-STM have driven substantial advances in catalysis research in recent years [118,119], such as alloy surface segregation phenomenon, distribution of active sites in single-atom catalysts, identification of oxide defect sites and adsorption/desorption dynamics of catalytic reactants [51,120,121,122]. With high resolution and in situ characterization capabilities, STM provides fundamental insights into surface structure and active sites of catalysts, and reaction mechanisms at the molecular level. Berndt et al. studied the adsorption of C_60_ on Au(111), finding that 49 of C_60_ molecules formed a Si(111)-7 × 7-like structure, as shown in Figure 13 [123]. They proposed a model that substrate-induced moiré-effect-like weeny orientation changes of C_60_ molecules allow the √589 × √589 R14.5° superlattice to offer epitaxial sites. These sites optimize intermolecular interactions, which is key for the superlattice’s stability.

**Figure 14:**
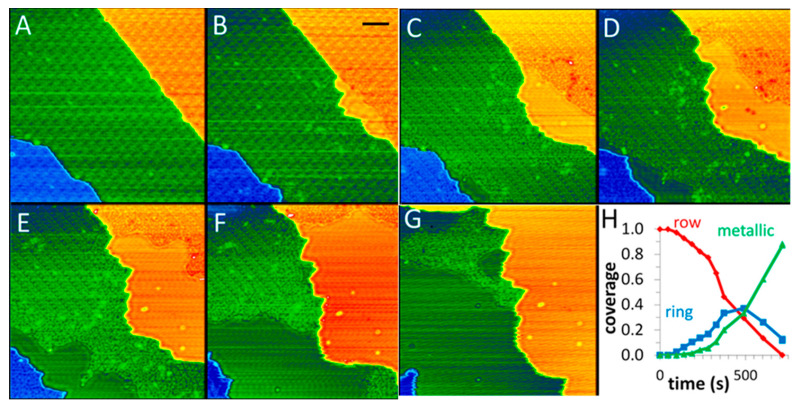
(**A**–**G**) STM snapshots during the reduction in a Cu_2_O film under 10 mTorr CO at 300 K after 46, 281, 374, 490, 603, 715, and 828 s. (**H**) Phase coverages are plotted as a function of CO exposure [50]. The color in the images corresponds to height contrast; U = 1.1 V, and I = 0.83 nA; scale bar = 5 nm.

In a case of Cu_2_O thin film [50], Baber et al. used NAP-STM to study the evolution process of Cu_2_O/Cu(111) in a CO atmosphere over time. Figure 14A depicts a fully oxidized Cu_2_O surface. Upon exposure to a CO atmosphere for 281 s, metallic Cu domains emerge at the step edges (Figure 14B). Progressive expansion of these metallic regions occurs with prolonged CO exposure, as shown in Figure 14C–G. The results reveal the continuous growth of metallic Cu domains along both step edges during reduction. This evolution indicates the directional migration of Cu atoms to step edges, driving phase segregation between oxide and metallic components to ultimately form metallic Cu steps.

## 4. Summary and Outlook

In summary, binary noble metal alloy catalysts have always been a hot topic in the field of heterogeneous catalysis. They hold the unique effects between two metals compared to corresponding single-metal catalysts, such as crystalline surface effects, synergistic effects, ligand effects, stain effects, and combinations thereof, which could tune the surface reactivity for enabling them to achieve superior catalytic activity, selectivity, and stability. The prospects of using coupled in situ imaging (TEM and STM) and in situ spectroscopy techniques to distinguish the dynamic correlation between structure and activity of alloy catalysts have been summarized above. The systematic framework for understanding the dynamic structural evolution of alloy catalysts under reactive gas environments was provided. We delve into fundamental phenomena such as surface segregation, phase separation, lattice transformation, and alloying processes. Despite there having been remarkable advances in the controlled syntheses, catalytic performances, and in situ characterizations of bimetallic alloy catalysts in recent years, following research efforts are still needed in the following aspects.

(1) **Improving the structural stability of alloy catalysts under reaction conditions.** Alloy catalysts, when subjected to high temperatures or prolonged reaction conditions, are prone to structural degradations such as particle sintering, and surface coarsening, which inevitably leads to a decline in catalytic activity and selectivity. Consequently, enhancing the stability the active structure of alloy catalysts remains a critical research priority during reaction.
(2) **Overcoming in situ TEM resolution and sensitivity limitations caused by environments and electron beam effects.** When conducting in situ imaging characterizations, the clear differences between the experimental environments and the actual working conditions of catalysts must be carefully considered. These differences may include the reactor dimensions, gas pressure, flow rate, and other parameters. In in situ TEM, gas molecule scattering and the window membrane in gas cell holders can adversely affect imaging quality. Moreover, electron beam irradiation can induce structural modifications in catalysts through the electron transfer process and localized heating effects. Taking these factors into account, in situ TEM techniques still require further advancements in both resolution and sensitivity, particularly in achieving higher temporal and spatial resolution, to more accurately capture the dynamic structural evolution of alloy catalysts under realistic operating conditions. Bridging the gap between the model in situ studies and industrial conditions now remains a key direction for the predictive design of active catalysts.
(3) **Developing the advanced and intelligentized methodology for automated tracking of atomic or nanoparticle trajectories.** In situ TEM experiments of catalysts can generate massive volumes of image and video data, which is used to capture dynamic structural and compositional changes at high temporal and spatial resolution. The enormous quantity and diversity of these datasets often exceed the capacity of conventional manual analysis methods, which are typically time-consuming and prone to the existence of subjective bias. Therefore, developing fast, accurate, and efficient data processing methodologies are needed, such as machine-learning-assisted image recognition and advanced image recognition algorithms for the automatic tracking of atomic or nanoparticle trajectories.

## Data Availability

No new data were created or analyzed in this study. Data sharing is not applicable to this article.

## Supplementary Materials

The following supporting information can be downloaded at https://www.mdpi.com/article/10.3390/nano15231828/s1. Table S1: Gas-mediated dynamic structure evolution of alloy catalysts under different atmosphere conditions.

## References

[1] Cao L., Lu J. (2020). Atomic-scale engineering of metal–oxide interfaces for advanced catalysis using atomic layer deposition. Catal. Sci. Technol..

[2] Vesborg P.C.K., Jaramillo T.F. (2012). Addressing the terawatt challenge: Scalability in the supply of chemical elements for renewable energy. RSC Adv..

[3] Wang X., Ruditskiy A., Xia Y.N. (2016). Rational design and synthesis of noble-metal nanoframes for catalytic and photonic applications. Natl. Sci. Rev..

[4] Wang H., Zhou S., Gilroy K.D., Cai Z., Xia Y. (2017). Icosahedral nanocrystals of noble metals: Synthesis and applications. Nano Today.

[5] Nakaya Y., Furukawa S. (2023). Catalysis of alloys: Classification, principles, and design for a variety of materials and reactions. Chem. Rev..

[6] Bera P., Hegde M.S. (2015). Noble metal ions in CeO_2_ and TiO_2_: Synthesis, structure and catalytic properties. RSC Adv..

[7] Rood S., Eslava S., Manigrasso A., Bannister C. (2019). Recent advances in gasoline three-way catalyst formulation: A review. Proc. Inst. Mech. Eng. Part D J. Automob. Eng..

[8] Hu Z., Wan C.Z., Lui Y.K., Dettling J., Steger J.J. (1996). Design of a novel Pd three-way catalyst: Integration of catalytic functions in three dimensions. Catal. Today.

[9] Yin X., Li S., Deng J., Zhao Y., Wang J., Chen Y. (2025). Efficient control of automotive emission by Pt-based and Rh-based three-way catalysts: The critical role of phase structure of ceria-zirconia support. Sep. Purif. Technol..

[10] Clauss D., Martin V., Nelayah J., Chattot R., Bordet P., Drnec J., Mirolo M., Dubau L., Maillard F. (2025). A Model approach to uncover the role of the IrO_x_ crystallographic structure and chemistry on OER activity and stability via annealing a sacrificial template. ACS Catal..

[11] Zhang T., Zheng P., Gao J., Liu X., Ji Y., Tian J., Zou Y., Sun Z., Hu Q., Chen G. (2024). Simultaneously activating molecular oxygen and surface lattice oxygen on Pt/TiO_2_ for low-temperature CO oxidation. Nat. Commun..

[12] Xu D., Jin Y., He B., Fang X., Chen G., Qu W., Xu C., Chen J., Ma Z., Chen L. (2024). Electronic communications between active sites on individual metallic nanoparticles in catalysis. Nat. Commun..

[13] Aso R., Tamaoka T., Yoshida H., Hojo H., Sano H., Midoh Y., Einaga H., Tanigaki T., Murakami Y. (2025). Direct visualization of surface structure and charge states of ceria-supported gold catalysts under redox conditions. Adv. Sci..

[14] Li H., Abdelgaid M., Paudel J.R., Holzapfel N.P., Augustyn V., McKone J.R., Mpourmpakis G., Crumlin E.J. (2025). Operando unveiling of hydrogen spillover mechanisms on tungsten oxide surfaces. J. Am. Chem. Soc..

[15] Duan C., Liu J., Li Z., Shi R., Zhao J., Waterhouse G.I.N., Wen X.D., Zhang L.P., Wu L.Z., Zhang T. (2025). Efficient photocatalytic propane direct dehydrogenation to propylene over PtO_2_ clusters. Adv. Mater..

[16] Wang J.Q., Song L.J., Huo J.T., Gao M., Zhang Y. (2024). Designing advanced amorphous/nanocrystalline alloys by controlling the energy state. Adv. Mater..

[17] He C., Gong Y., Li S., Wu J., Lu Z., Li Q., Wang L., Wu S., Zhang J. (2024). Single-atom alloys materials for CO_2_ and CH_4_ catalytic conversion. Adv. Mater..

[18] Ruban A., Hammer B., Stoltze P., Skriver H.L., Nørskov J.K. (1997). Surface electronic structure and reactivity of transition and noble metals. J. Mol. Catal. A Chem..

[19] Rossmeisl J., Qu Z.W., Zhu H., Kroes G.J., Nørskov J.K. (2007). Electrolysis of water on oxide surfaces. J. Electroanal. Chem..

[20] Bligaard T., Nørskov J.K. (2007). Ligand effects in heterogeneous catalysis and electrochemistry. Electrochim. Acta.

[21] Xin H., Holewinski A., Schweitzer N., Nikolla E., Linic S. (2012). Electronic structure engineering in heterogeneous catalysis: Identifying novel alloy catalysts based on rapid screening for materials with desired electronic properties. Top Catal..

[22] Wei Z., Yu S., Li C. (2024). Research development of anti-CO poisoning in electrocatalytic methanol oxidation processes: A review. Catal. Sci. Technol..

[23] Liu Y., Chi M., Mazumder V., More K.L., Soled S., Henao J.D., Sun S. (2011). Composition-controlled synthesis of bimetallic PdPt nanoparticles and their electro-oxidation of methanol. Chem. Mater..

[24] Li B., Higgins D.C., Zhu S., Li H., Wang H., Ma J., Chen Z. (2012). Highly active Pt–Ru nanowire network catalysts for the methanol oxidation reaction. Catal. Commun..

[25] Hwang S.J., Kim S.K., Lee J.G., Lee S.C., Jang J.H., Kim P., Lim T.H., Sung Y.E., Yoo S.J. (2012). Role of electronic perturbation in stability and activity of Pt-based alloy nanocatalysts for oxygen reduction. J. Am. Chem. Soc..

[26] Zhao J.W., Wang H.Y., Feng L., Zhu J.Z., Liu J.X., Li W.X. (2024). Crystal-phase engineering in heterogeneous catalysis. Chem. Rev..

[27] Zhuang J., Wang D. (2023). Recent advances of single-atom alloy catalyst: Properties, synthetic methods and electrocatalytic applications. Mater. Today Catal..

[28] Lin F., Li M., Zeng L., Luo M., Guo S. (2023). Intermetallic nanocrystals for fuel-cells-based electrocatalysis. Chem. Rev..

[29] Zhang X., Sun Z., Jin R., Zhu C., Zhao C., Lin Y., Guan Q., Cao L., Wang H., Li S. (2023). Conjugated dual size effect of core-shell particles synergizes bimetallic catalysis. Nat. Commun..

[30] Tritsaris G.A., Greeley J., Rossmeisl J., Nørskov J.K. (2011). Atomic-scale modeling of particle size effects for the oxygen reduction reaction on Pt. Catal. Lett..

[31] Yang X.F., Wang A., Qiao B., Li J., Liu J., Zhang T. (2013). Single-atom catalysts: A new frontier in heterogeneous catalysis. Acc. Chem. Res..

[32] Shao M., Peles A., Shoemaker K. (2011). Electrocatalysis on platinum nanoparticles: Particle size effect on oxygen reduction reaction activity. Nano Lett..

[33] Sheng T., Tian N., Zhou Z.-Y., Lin W.-F., Sun S.-G. (2017). Designing Pt-based electrocatalysts with high surface energy. ACS Energy Lett..

[34] Zhang J., Vukmirovic M.B., Sasaki K., Nilekar A.U., Mavrikakis M., Adzic R.R. (2005). Mixed-metal Pt monolayer electrocatalysts for enhanced oxygen reduction kinetics. J. Am. Chem. Soc..

[35] Zhu J., Xu L., Lyu Z., Xie M., Chen R., Jin W., Mavrikakis M., Xia Y. (2021). Janus Nanocages of Platinum-Group Metals and Their Use as Effective Dual-Electrocatalysts. Angew. Chem. Int. Ed..

[36] Asano M., Kawamura R., Sasakawa R., Todoroki N., Wadayama T. (2016). Oxygen reduction reaction activity for strain-controlled Pt-based model alloy catalysts: Surface strains and direct electronic effects induced by alloying elements. ACS Catal..

[37] Zhang Y., Ye K., Liu Q., Qin J., Jiang Q., Yang B., Yin F. (2022). Ni^2+^-directed anisotropic growth of PtCu nested skeleton cubes boosting electroreduction of oxygen. Adv. Sci..

[38] Zhang Y.F., Qin J., Leng D.Y., Liu Q.R., Xu X.Y., Yang B., Yin F. (2021). Tunable strain drives the activity enhancement for oxygen reduction reaction on Pd@Pt core-shell electrocatalysts. J. Power Sources.

[39] Zhang Y., Ye K., Gu Q., Jiang Q., Qin J., Leng D., Liu Q., Yang B., Yin F. (2021). Optimized oxygen reduction activity by tuning shell component in Pd@Pt-based core-shell electrocatalysts. J. Colloid Interface Sci..

[40] Mahbub M.A.A., Junqueira J.R.C., Wang X., Zhang J., Dieckhöfer S., Seisel S., Das D., Schuhmann W. (2023). Dynamic transformation of functionalized bismuth to catalytically active surfaces for CO_2_ reduction to formate at high current densities. Adv. Funct. Mater..

[41] Tao F., Salmeron M. (2024). Surface restructuring and predictive design of heterogeneous catalysts. Science.

[42] Chao H.Y., Venkatraman K., Moniri S., Jiang Y., Tang X., Dai S., Gao W., Miao J., Chi M. (2023). In situ and emerging transmission electron microscopy for catalysis research. Chem. Rev..

[43] Xiao M., Sun H., Meng Y., Zhu F. (2024). Advances of in situ transmission electron microscopy research on gas phase catalyst particles. Catal. Sci. Technol..

[44] Li S., Wang G., Lv H., Lin Z., Liang J., Liu X., Wang Y.G., Huang Y., Wang G., Li Q. (2024). Constructing gradient orbital coupling to induce reactive metal-support interaction in Pt-carbide electrocatalysts for efficient methanol oxidation. J. Am. Chem. Soc..

[45] Jalil A., Happel E.E., Cramer L., Hunt A., Hoffman A.S., Waluyo I., Montemore M.M., Christopher P., Sykes E.C.H. (2025). Nickel promotes selective ethylene epoxidation on silver. Science.

[46] Crozier P.A., Leibovich M., Haluai P., Tan M., Thomas A.M., Vincent J., Mohan S., Marcos Morales A., Kulkarni S.A., Matteson D.S. (2025). Visualizing nanoparticle surface dynamics and instabilities enabled by deep denoising. Science.

[47] Zhou S., Shi J., Liu S., Li G., Pei F., Chen Y., Deng J., Zheng Q., Li J., Zhao C. (2023). Visualizing interfacial collective reaction behaviour of Li-S batteries. Nature.

[48] Salvato M., Crescenzi M., Scagliotti M., Castrucci P., Boninelli S., Caruso G.M., Liu Y., Mikkelsen A., Timm R., Nahas S. (2022). Nanometric moire stripes on the surface of Bi_2_Se_3_ topological insulator. ACS Nano.

[49] Feng Q., Zhu C., Sheng G., Sun T., Li Y., Zhu Y. (2023). Four-dimensional scanning transmission electron microscopy: From material microstructures to physicochemical properties. Acta Phys. Chim. Sin..

[50] Baber A.E., Xu F., Dvorak F., Mudiyanselage K., Soldemo M., Weissenrieder J., Senanayake S.D., Sadowski J.T., Rodriguez J.A., Matolin V. (2013). In situ imaging of Cu_2_O under reducing conditions: Formation of metallic fronts by mass transfer. J. Am. Chem. Soc..

[51] Fu Q., Li W.X., Yao Y., Liu H., Su H.Y., Ma D., Gu X.K., Chen L., Wang Z., Zhang H. (2010). Interface-confined ferrous centers for catalytic oxidation. Science.

[52] Tao F., Grass M.E., Zhang Y., Butcher D.R., Renzas J.R., Liu Z., Chung J.Y., Mun B.S., Salmeron M., Somorjai G.A. (2008). Reaction-driven restructuring of Rh-Pd and Pt-Pd core-shell nanoparticles. Science.

[53] Andersson K.J., Calle-Vallejo F., Rossmeisl J., Chorkendorff I. (2009). Adsorption-driven surface segregation of the less reactive alloy component. J. Am. Chem. Soc..

[54] Ma T., Fu Q., Su H.Y., Liu H.Y., Cui Y., Wang Z., Mu R.T., Li W.X., Bao X.H. (2009). Reversible structural modulation of Fe-Pt bimetallic surfaces and its effect on reactivity. ChemPhysChem.

[55] Kim H.Y., Henkelman G. (2013). CO Adsorption-driven surface segregation of Pd on Au/Pd bimetallic surfaces: Role of defects and effect on CO oxidation. ACS Catal..

[56] McCue A.J., Anderson J.A. (2015). CO induced surface segregation as a means of improving surface composition and enhancing performance of CuPd bimetallic catalysts. J. Catal..

[57] Wu C.H., Liu C., Su D., Xin H.L., Fang H.-T., Eren B., Zhang S., Murray C.B., Salmeron M.B. (2018). Bimetallic synergy in cobalt–palladium nanocatalysts for CO oxidation. Nat. Catal..

[58] Xu C., Han S., Zhao Q., Liu S., Liu W., Li Y., Shen W. (2024). Structure evolution of Cu_3_Pd single-particles under CO_2_ hydrogenation. Chem. Eng. J..

[59] Sandoval-Diaz L., Cruz D., Vuijk M., Ducci G., Hävecker M., Jiang W., Plodinec M., Hammud A., Ivanov D., Götsch T. (2024). Metastable nickel–oxygen species modulate rate oscillations during dry reforming of methane. Nat. Catal..

[60] Zhang R.P., He B., Liu X., Lu A.H. (2023). Hydrogen spillover-driven dynamic evolution and migration of iron oxide for structure regulation of versatile magnetic nanocatalysts. J. Am. Chem. Soc..

[61] Usoltsev O., Stoian D., Skorynina A., Kozyr E., Njoroge P.N., Pellegrini R., Groppo E., van Bokhoven J.A., Bugaev A. (2024). restructuring of palladium nanoparticles during oxidation by molecular oxygen. Small.

[62] Lee J., Christopher P. (2024). Does H_2_ temperature-programmed reduction always probe solid-state redox chemistry? the case of Pt/CeO_2_. Angew. Chem. Int. Ed..

[63] Ricchebuono A., Vottero E., Bonavia D., Lazzarini P., Pellegrini R., Crocellà V., Porcaro N.G., Checchia S., Ferri D., Piovano A. (2024). CO-induced dynamic behavior of Al_2_O_3_-supported Pd nanoparticles at room temperature. ACS Catal..

[64] Dai S., You Y., Zhang S., Cai W., Xu M., Xie L., Wu R., Graham G.W., Pan X. (2017). In situ atomic-scale observation of oxygen-driven core-shell formation in Pt_3_Co nanoparticles. Nat. Commun..

[65] Cramer L.A., Liu Y., Deshlahra P., Sykes E.C.H. (2020). Dynamic restructuring induced oxygen activation on AgCu near-surface alloys. J. Phys. Chem. Lett..

[66] van Spronsen M.A., Daunmu K., O’Connor C.R., Egle T., Kersell H., Oliver-Meseguer J., Salmeron M.B., Madix R.J., Sautet P., Friend C.M. (2018). Dynamics of surface alloys: Rearrangement of Pd/Ag(111) induced by CO and O_2_. J. Phys. Chem. C.

[67] Luneau M., Guan E., Chen W., Foucher A.C., Marcella N., Shirman T., Verbart D.M.A., Aizenberg J., Aizenberg M., Stach E.A. (2020). Enhancing catalytic performance of dilute metal alloy nanomaterials. Commun. Chem..

[68] Niu Y., Wang Y., Chen J., Li S., Huang X., Willinger M.G., Zhang W., Liu Y., Zhang B. (2022). Patterning the consecutive Pd_3_ to Pd_1_ on Pd_2_Ga surface via temperature-promoted reactive metal-support interaction. Sci. Adv..

[69] Zhou C., Ngan H.T., Lim J.S., Darbari Z., Lewandowski A., Stacchiola D.J., Kozinsky B., Sautet P., Boscoboinik J.A. (2022). Dynamical study of adsorbate-induced restructuring kinetics in bimetallic catalysts using the PdAu(111) model system. J. Am. Chem. Soc..

[70] Ouyang M., Papanikolaou K.G., Boubnov A., Hoffman A.S., Giannakakis G., Bare S.R., Stamatakis M., Flytzani-Stephanopoulos M., Sykes E.C.H. (2021). Directing reaction pathways via in situ control of active site geometries in PdAu single-atom alloy catalysts. Nat. Commun..

[71] Gibson E.K., Beale A.M., Catlow C.R.A., Chutia A., Gianolio D., Gould A., Kroner A., Mohammed K.M.H., Perdjon M., Rogers S.M. (2015). Restructuring of AuPd nanoparticles studied by a combined XAFS/DRIFTS approach. Chem. Mater..

[72] Hansen C., Zhou W., Brack E., Wang Y., Wang C., Paterson J., Southouse J., Copéret C. (2024). Decoding the promotional effect of iron in bimetallic Pt–Fe-nanoparticles on the low temperature reverse water–gas shift reaction. J. Am. Chem. Soc..

[73] Marcella N., Lim J.S., Plonka A.M., Yan G., Owen C.J., van der Hoeven J.E.S., Foucher A.C., Ngan H.T., Torrisi S.B., Marinkovic N.S. (2022). Decoding reactive structures in dilute alloy catalysts. Nat. Commun..

[74] Pielsticker L., Zegkinoglou I., Divins N.J., Mistry H., Chen Y.T., Kostka A., Boscoboinik J.A., Cuenya B.R. (2018). Segregation phenomena in size-selected bimetallic CuNi nanoparticle catalysts. J. Phys. Chem. B.

[75] Tang M., Yuan W., Ou Y., Li G., You R., Li S., Yang H., Zhang Z., Wang Y. (2020). Recent progresses on structural reconstruction of nanosized metal catalysts via controlled-atmosphere transmission electron microscopy: A review. ACS Catal..

[76] Zhou C., Zhang Y.F., Li B., Yang B., Li L. (2024). Resolving the active role of isolated transition metal species in Ni-based catalysts for dry reforming of methane. ACS Catal..

[77] Zhang X., Han S., Zhu B., Zhang G., Li X., Gao Y., Wu Z., Yang B., Liu Y., Baaziz W. (2020). Reversible loss of core–shell structure for Ni–Au bimetallic nanoparticles during CO_2_ hydrogenation. Nat. Catal..

[78] Jiang Y., Wong Z.M., Yan H., Tan T.L., Mirsaidov U. (2025). Revealing Multistep Phase Separation in Metal Alloy Nanoparticles with In Situ Transmission Electron Microscopy. ACS Nano.

[79] Kim T.-S., Choi H., Kim D., Song H.C., Oh Y., Jeong B., Lee J., Kim K.-J., Shin J.W., Byon H.R. (2023). Catalytic boosting on AuCu bimetallic nanoparticles by oxygen-induced atomic restructuring. Appl. Catal. B Environ..

[80] Foucher A.C., Marcella N., Lee J.D., Rosen D.J., Tappero R., Murray C.B., Frenkel A.I., Stach E.A. (2021). Structural and valence state modification of cobalt in CoPt nanocatalysts in redox conditions. ACS Nano.

[81] Yao Y., Goodman D.W. (2014). In situ IR spectroscopic studies of Ni surface segregation induced by CO adsorption on Cu-Ni/SiO_2_ bimetallic catalysts. Phys. Chem. Chem. Phys..

[82] Wang Q., Xia G.-J., Zhao Z.L., Zhu Y., Shi X., Huang L., Wang Y.-G., Gu M. (2020). Atomic origin of CO-interaction effect of PtPb@Pt catalyst revealed by in situ environmental transmission electron microscopy. Nano Energy.

[83] Silva T.A.G., Teixeira-Neto É., Borges L.R., Neves-Garcia T., Braga A.H., Rossi L.M. (2023). From AuPd nanoparticle alloys towards core-shell motifs with enhanced alcohol oxidation activity. ChemCatChem.

[84] Foucher A.C., Owen C.J., Shirman T., Aizenberg J., Kozinsky B., Stach E.A. (2022). Atomic-Scale STEM analysis shows structural changes of Au–Pd nanoparticles in various gaseous environments. J. Phys. Chem. C.

[85] Zhang Y., Li H., Liu F., Li M., Zhang Y., Cai J., Li Y., Yang F., Yin F., Lu J. (2024). Revealing dynamics and competitive mechanism of gas-induced surface segregation of PdFe_0.08_ dilute alloy by multi-dimensional imaging. J. Phys. Chem. Lett..

[86] Beaumont S.K., Alayoglu S., Pushkarev V.V., Liu Z., Kruse N., Somorjai G.A. (2013). Exploring surface science and restructuring in reactive atmospheres of colloidally prepared bimetallic CuNi and CuCo nanoparticles on SiO_2_ in situ using ambient pressure X-ray photoelectron spectroscopy. Faraday Discuss.

[87] Cao Z., Dong Z., Yang S., Cui R., Zhang L., Chen X., Luo L. (2024). Phase Separation of CuPd Alloy Nanocatalysts in CO Oxidation. ACS Nano.

[88] Jiang Y., Lim A.M.H., Yan H., Zeng H.C., Mirsaidov U. (2023). Phase segregation in PdCu alloy nanoparticles during CO oxidation reaction at atmospheric pressure. Adv. Sci..

[89] Li Y., Guo L., Du M., Tian C., Zhao G., Liu Z., Liang Z., Hou K., Chen J., Liu X. (2024). Unraveling distinct effects between CuO_x_ and PtCu alloy sites in Pt-Cu bimetallic catalysts for CO oxidation at different temperatures. Nat. Commun..

[90] Song Y., Kim D., Hong S., Kim T.-S., Kim K.-J., Park J.Y. (2023). Bimetallic synergy from a reaction-driven metal oxide–metal interface of Pt–Co bimetallic nanoparticles. ACS Catal..

[91] Ghosh T., Liu X., Sun W., Chen M., Liu Y., Li Y., Mirsaidov U. (2022). Revealing the origin of low-temperature activity of Ni-Rh nanostructures during CO oxidation reaction with operando TEM. Adv. Sci..

[92] Jiang Y., Duchamp M., Ang S.J., Yan H., Tan T.L., Mirsaidov U. (2023). Dynamics of the fcc-to-bcc phase transition in single-crystalline PdCu alloy nanoparticles. Nat. Commun..

[93] Liu S., Li Y., Yu X., Han S., Zhou Y., Yang Y., Zhang H., Jiang Z., Zhu C., Li W.X. (2022). Tuning crystal-phase of bimetallic single-nanoparticle for catalytic hydrogenation. Nat. Commun..

[94] Qiu Y., Xin L., Li Y., McCrum I.T., Guo F., Ma T., Ren Y., Liu Q., Zhou L., Gu S. (2018). BCC-phased PdCu alloy as a highly active electrocatalyst for hydrogen oxidation in alkaline electrolytes. J. Am. Chem. Soc..

[95] Lim J., Cullen D.A., Stavitski E., Lee S.W., Hatzell M.C. (2023). Atomically ordered PdCu electrocatalysts for selective and stable electrochemical nitrate reduction. ACS Energy Lett..

[96] Jia L., Sun M., Xu J., Zhao X., Zhou R., Pan B., Wang L., Han N., Huang B., Li Y. (2021). Phase-dependent electrocatalytic CO_2_ reduction on Pd_3_Bi nanocrystals. Angew. Chem. Int. Ed..

[97] Ji Y., Chen Z., Wei R., Yang C., Wang Y., Xu J., Zhang H., Guan A., Chen J., Sham T.-K. (2022). Selective CO-to-acetate electroreduction via intermediate adsorption tuning on ordered Cu–Pd sites. Nat. Catal..

[98] Tong W., Huang B., Wang P., Li L., Shao Q., Huang X. (2020). Crystal-phase-engineered PdCu electrocatalyst for enhanced ammonia synthesis. Angew. Chem. Int. Ed..

[99] Chen H., Li L., Zhao Z.J., Yang B., Zhang Y., Liu X., Gu Q., Yu Z., Yang X., Gong J. (2024). Co-infiltration and dynamic formation of Pd_3_ZnC_x_ intermetallic carbide by syngas boosting selective hydrogenation of acetylene. Nat. Commun..

[100] Sun Q., Liu X., Gu Q., Sun Z., Wang H., Cao L., Xu Y., Li S., Yang B., Wei S. (2024). Breaking the conversion-selectivity trade-off in methanol synthesis from CO_2_ using dual intimate oxide/metal interfaces. J. Am. Chem. Soc..

[101] Liu X., Gu Q., Zhang Y., Xu X., Wang H., Sun Z., Cao L., Sun Q., Xu L., Wang L. (2023). Atomically thick oxide overcoating stimulates low-temperature reactive metal-support interactions for enhanced catalysis. J. Am. Chem. Soc..

[102] Ouvrard A., Alyabyeva N., Zakaria A.M., Yuan K., Dablemont C., Lazzari R., Charra F., Bourguignon B. (2024). Change of composition and surface plasmon resonance of Pd/Au core/shell nanoparticles triggered by CO adsorption. J. Chem. Phys..

[103] Yang F., Wang Y., Cui Y., Yang X., Zhu Y., Weiss C.M., Li M., Chen G., Yan Y., Gu M.D. (2023). Sub-3 nm Pt@Ru toward outstanding hydrogen oxidation reaction performance in alkaline media. J. Am. Chem. Soc..

[104] Han J., Yang J., Zhang Z., Jiang X., Liu W., Qiao B., Mu J., Wang F. (2023). Strong metal–support interaction facilitated multicomponent alloy formation on metal oxide support. J. Am. Chem. Soc..

[105] Zhang X., Shi W., Li Y., Zhao W., Han S., Shen W. (2023). Pt_3_Ti intermetallic alloy formed by strong metal–support interaction over Pt/TiO_2_ for the selective hydrogenation of acetophenone. ACS Catal..

[106] Niu Z., Chen S., Yu Y., Lei T., Dehestani A., Schierle-Arndt K., Yang P. (2020). Morphology-controlled transformation of Cu@Au core-shell nanowires into thermally stable Cu_3_Au intermetallic nanowires. Nano Res..

[107] Du P., Zhang Y., Qi R., Gu Q., Xu X., Wang A., Zhu B., Yang B., Zhang T. (2025). Domino effect of catalysis: Coherence between reaction network and catalyst restructuring accelerating surface carburization for CO_2_ hydrogenation. J. Am. Chem. Soc..

[108] Reddy K.P., Kim D., Hong S., Kim K.J., Ryoo R., Park J.Y. (2023). Tuning CO_2_ hydrogenation selectivity through reaction-driven restructuring on Cu-Ni bimetal catalysts. ACS Appl. Mater. Interfaces.

[109] Guo J., Wang Z., Gao T., Wang Z. (2024). Experimental and theoretical study of Pd-Pt/In_2_O_3_ bimetallic catalysts for enhancing methanol production from CO_2_. Chem. Eng. J..

[110] De Broglie L. (1923). Waves and quanta. Nature.

[111] Knoll M., Ruska E. (1932). Das elektronenmikroskop. Z. Phys..

[112] Tang M., Li S., Chen S., Ou Y., Hiroaki M., Yuan W., Zhu B., Yang H., Gao Y., Zhang Z. (2021). Facet-dependent oxidative strong metal-support interactions of palladium-TiO_2_ determined by in situ transmission electron microscopy. Angew. Chem. Int. Ed..

[113] Yuan W., Zhu B., Fang K., Li X.Y., Hansen T.W., Ou Y., Yang H., Wagner J.B., Gao Y., Wang Y. (2021). In situ manipulation of the active Au-TiO_2_ interface with atomic precision during CO oxidation. Science.

[114] Gu J.Y., Cai Z.F., Wang D., Wan L.J. (2016). Single-molecule imaging of iron-phthalocyanine-catalyzed oxygen reduction reaction by in situ scanning tunneling microscopy. ACS Nano.

[115] Valls Mascaró F., Koper M.T.M., Rost M.J. (2024). Step bunching instability and its effects in electrocatalysis on platinum surfaces. Nat. Catal..

[116] Yuan W., Zhu B., Li X.Y., Hansen T.W., Ou Y., Fang K., Yang H., Zhang Z., Wagner J.B., Gao Y. (2020). Visualizing H_2_O molecules reacting at TiO_2_ active sites with transmission electron microscopy. Science.

[117] Yun H., Zhang D., Birol T., Wang J.P., Mkhoyan K.A. (2023). Structural anisotropy-driven atomic mechanisms of phase transformations in the Pt-Sn system. Nano Lett..

[118] Binnig G., Rohrer H., Gerber C., Weibel E. (1982). Surface studies by scanning tunneling microscopy. Phys. Rev. Lett..

[119] Laegsgaard E., Österlund L., Thostrup P., Rasmussen P.B., Stensgaard I., Besenbacher F. (2001). A high-pressure scanning tunneling microscope. Rev. Sci. Instrum..

[120] Hulsken B., Van Hameren R., Gerritsen J.W., Khoury T., Thordarson P., Crossley M.J., Rowan A.E., Nolte R.J., Elemans J.A., Speller S. (2007). Real-time single-molecule imaging of oxidation catalysis at a liquid-solid interface. Nat. Nanotechnol..

[121] Kyriakou G., Boucher M.B., Jewell A.D., Lewis E.A., Lawton T.J., Baber A.E., Tierney H.L., Flytzani-Stephanopoulos M., Sykes E.C. (2012). Isolated metal atom geometries as a strategy for selective heterogeneous hydrogenations. Science.

[122] Vestergaard E.K., Vang R.T., Knudsen J., Pedersen T.M., An T., Laegsgaard E., Stensgaard I., Hammer B., Besenbacher F. (2005). Adsorbate-induced alloy phase separation: A direct view by high-pressure scanning tunneling microscopy. Phys. Rev. Lett..

[123] Schull G., Berndt R. (2007). Orientationally ordered (7×7) superstructure of C_60_ on Au(111). Phys. Rev. Lett..

