# Supplementary material for: Gas-Mediated Dynamic Structure Evolution of Bimetallic Alloy Catalysts
Source: Nanomaterials (Basel). 2025 Dec 3;15(23):1828. doi: 10.3390/nano15231828 (PMC12693368; doi:10.3390/nano15231828)
Supplement: Supplementary file 1 [file nanomaterials-15-01828-s001.zip › nanomaterials-3984361-supplementary.pdf]

Gas-mediated dynamic structure evolution of bimetallic alloy catalysts

Yafeng Zhang [1,2], Pengfei Du [2] and Bing Yang [2,*]

1 New Energy Materials and Physics Laboratory, School of Physics, Ningxia University, Yinchuan 750021, China

2 CAS Key Laboratory of Science and Technology on Applied Catalysis, Dalian Institute of Chemical Physics, Chinese Academy of Sciences, Dalian 116023, China

Table S1. Gas-mediated dynamic structure evolution of alloy catalysts under different atmosphere conditions.

**Catalyst**	**O_2**	**H_2**	**CO**	**CO_2+H_2**	**CO+O_2**	**Ref.**	**Structural types**
Pt_3Co alloys	Pt surface enrichment	-	-	-	-	60	Gas-mediated surface reconstruction (Surface atom enrichment)
Ni-Ag surface alloys	Ni surface enrichment	-	-	-	-	45	
PdAg@Ag core–shell	Pd surface enrichment	-	-	-	-	62	
PdAu alloys	PdO-rich surface	-	-	-	-	63	
Pd_3Cu alloys	-	Cu surface enrichment	-	-	-	58	
PdAu alloys	-	-	Pd surface enrichment	-	-	63	
Pd_2Ga alloys	-	Surface Pd trimers	-	-	-	64	

PdAu alloys	-	-	Pd surface enrichment	-	-	65	Gas-mediated surface reconstruction (Surface atom enrichment)
PdAu alloys	-	-	Pd surface enrichment	-	-	66	
PdFe alloys	-	-	-	surface Fe oxidation and reduction	-	68	
Ni@Au core–shell	-	-	-	NiAu surface alloying	-	73	
AuCu alloys	CuO_x/Au interface	-	-	-	-	75	Gas-mediated surface segregation (Separated phase)
Co_2Pt_3 alloys	CoPt-Co_3O_4 interface	-	-	-	-	76	
CuNi alloys	-	-	Surface Ni segregation	-	-	77	
PtPb@Pt core–shell	-	-	PtPb-$Pb(CO)_4$ interface	-	-	78	
PdCu/CeO_2	-	-	-	-	PdCu-Cu_2O interface	83	

PdCu alloys	-	-	-	-	Pd-CuO interface	84	Gas-mediated surface segregation (Separated phase)
PtCu alloys	-	-	-	-	PtCu-CuOx interface	85	
PtCo alloys	-	-	-	-	Pt-CoO interface	86	
PdCu alloys	-	fcc-to-bcc phase transition	-	-	-	88	Gas-mediated dynamic phase transition
PdCu alloys	-	Disordered to ordered transformation	-	-	-	92	
PdCu alloys	-	Disordered to ordered transformation	-	-	-	93	
$PdO/ZnO/Al_2O_3$	-	Pd_3ZnC_x phase		-	-	95	
$PdCu@InO_x$	-	PdIn alloys		-	-	96	
$Pd@Ga_2O_3$	-	Pd_2Ga	-	-	-	97	

Pd@Au core–shell	-	-	PdAu alloying shell	-	-	98	Gas-mediated alloying
Pt@Ru core–shell	-	PtRu alloys	-	-	-	99	
Pt/TiO_2	-	Pt_3Ti alloys	-	-	-	101	
Cu@Au core–shell	-	CuAu alloys	-	-	-	102	
Pd/FeO_x	-	-	-	Pd_3Fe/FeO_x	-	103	
Cu@Ni core–shell	-	-	-	Cu-Ni alloys	-	104	
